# A Comprehensive Analysis of Extraction Methods, Phytochemical Characterization and Neuroprotective Potential of Curcumin

**DOI:** 10.3390/nu18203343

**Published:** 2026-10-12

**Authors:** Denise Maria Dardano, Roberta Macrì, Maria Serra, Carmen Altomare, Giovanna Ritorto, Sara Ussia, Muhammad Mubeen Jamal Anwar, Ernesto Palma, Vincenzo Mollace

**Affiliations:** 1 Pharmacology Laboratory, Institute of Research for Food Safety and Health (IRC-FSH), Department of Health Sciences, University “Magna Graecia” of Catanzaro, 88100 Catanzaro, Italy; denisemaria.dardano@studenti.unicz.it (D.M.D.); carmen.altomare@studenti.unicz.it (C.A.); muhammadmubeenjamal.anwar@studenti.unicz.it (M.M.J.A.);; 2 Laboratory of Mass Spectrometry and Proteomics, Department of Experimental and Clinical Medicine, University “Magna Graecia” of Catanzaro, 88100 Catanzaro, Italy; 3 Veterinary Pharmacology Laboratory, Institute of Research for Food Safety and Health (IRC-FSH), Department of Health Sciences, University “Magna Graecia” of Catanzaro, 88100 Catanzaro, Italy; 4 Renato Dulbecco Institute, 88046 Lamezia Terme, Italy

**Keywords:** Curcumin, *Curcuma longa*, neuroinflammation, neurodegenerative disease, neuroimmune disease

## Abstract

Curcumin, a natural polyphenol derived from *Curcuma longa*, exhibits multi-targeted anti-inflammatory, antioxidant, and neuroprotective activities. This review aims to evaluate its extraction methodologies, synthetic pathways, and neuroprotective potential across preclinical and clinical paradigms, while addressing bioavailability barriers and commercial adulteration. In cellular models (BV-2, U87), curcumin inhibits TLR4/MyD88/NF-κB and NLRP3 inflammasome signaling, resulting in the downregulation of pro-inflammatory cytokines (IL-1β, TNF-α, IL-6) and enzymes (COX-2, iNOS), while simultaneously activating Nrf2/ARE antioxidant pathways. Moreover, it facilitates the polarization of neuroprotective M2 microglia. In animal models of ischemic stroke, traumatic brain injury, and Parkinson’s disease, curcumin reduces neuroinflammation, protects dopaminergic neurons, and maintains the integrity of the blood–brain barrier. Advanced nanocarriers such as PLGA nanoparticles, exosomes, and lipid complexes bypass rapid hepatic metabolism, thereby increasing brain bioavailability. Clinical data demonstrate that bioavailable formulations (Theracurmin, Longvida, CurQfen) could improve memory function and reduce cerebral amyloid and tau deposits in subjects without dementia and with mild cognitive impairment. Conversely, the use of unformulated curcumin results in inconsistent findings in trials for Alzheimer’s and Parkinson’s diseases owing to its poor systemic absorption. In conclusion, Curcumin might represent a promising multitarget neuroprotective agent; however, addressing pharmacokinetic limitations via bioavailable delivery systems and implementing rigorous quality control to prevent commercial adulteration are essential for effective clinical application.

## 1. Introduction

### 1.1. Curcumin Production: Methodologies and Extraction Paradigms

A sophisticated and integrated series of extraction, purification, and analytical characterization techniques is needed to isolate the principal bioactive curcuminoid, (1E,6E)-1,7-bis(4-hydroxy-3-methoxyphenyl)-1,6-heptadiene-3,5-dione, from the *Curcuma longa* plant material [1]. The sophisticated methodology has been precisely developed to address the inherent difficulties in processing plant tissues, incorporating a range of physicochemical techniques to optimize both the high-grade purity and the substantial recovery of target polyphenolic compounds from their intracellular confinement (Figure 1) [2,3,4].

#### 1.1.1. Enzyme-Assisted Extraction (EAE)

The methodology of EAE operates by utilizing specific hydrolytic enzymes, such as lipases, amylases, pectinases, amyloglucosidases, and proteases, to catalyze the degradation of complex plant cell wall polysaccharides [5,6]. The structural integrity of cellular architecture is compromised by targeted degradation, leading to the release of sequestered curcuminoids into the solvent [5,7]. Achieving optimal results in this extraction process necessitates careful control over essential factors, specifically the hydrogen ion concentration, temperature, enzyme quantity, and incubation period [8,9,10]. Specifically, the simultaneous use of α-amylase and amyloglucosidase has been reported to achieve a substantial curcuminoid recovery rate of around 4.1% [2].

#### 1.1.2. Supercritical Fluid Extraction (SFE)

Supercritical Fluid Extraction (SFE) utilizes the specialized state of carbon dioxide (CO_2_) when it exceeds its critical temperature and pressure [11]. In this supercritical phase, CO_2_ possesses properties that are a hybrid of gaseous and liquid states, thereby improving its diffusion and mass transfer efficiencies [12]. CO_2_ is favored due to its low critical temperature and environmental sustainability [13,14]; due to its intrinsic low polarity, polar co-solvents, such as ethanol or methanol, are frequently employed to augment the solubility of the comparatively hydrophobic curcumin molecule. A study by Pyo and Kim (2014) documented a yield of 31.07 mg/g with ethanol-modified supercritical CO_2_ [15]. In contrast, more intricate integrated systems employing Supercritical AntiSolvent (SAS) and Pressurized Liquid Extraction (PLE) techniques have reported curcuminoid yields as high as 7.6% [11,16].

#### 1.1.3. Microwave-Assisted Extraction (MAE)

The operation of Microwave-Assisted Extraction (MAE) is predicated on the precise interaction between microwave radiation (typically 300 MHz to 300 GHz) and the plant matrix [17], thereby instigating rapid, localized heating through dipole rotation and ionic conduction mechanisms [3,18]. Consequently, this thermomechanical stress causes substantial cellular damage, facilitating the rapid release of bioactive secondary metabolites into the extractant [19,20]. Comparative analyses demonstrate that MAE significantly enhances extraction efficiency and decreases energy consumption compared to conventional Soxhlet techniques, as evidenced by Singh et al.’s reported yield of 326.79 mg/g [21] and Fernandez-Marin et al. achieving an oil yield of 10.32% [22]. Furthermore, the synergistic application of MAE alongside enzymatic and ultrasonic methods has been shown to facilitate a curcuminoid recovery of 2.89% [23].

#### 1.1.4. Green Solvent-Based and Combined Strategies

The field of extraction science has progressively prioritized the use of eco-friendly solvents, including subcritical water, ionic liquids, and both Deep Eutectic Solvents (DESs) and Natural Deep Eutectic Solvents (NADESs) [24,25]. The modulation of thermodynamic variables [26] and the increased stability of curcumin [27,28] contribute to high recovery rates within these systems. Additionally, the synergistic effect derived from integrated methodologies, exemplified by the combination of ultrasound-assisted extraction and supercritical CO_2_, has been demonstrated to concurrently enhance the purity and overall recovery of the isolated curcuminoids [29,30].

#### 1.1.5. Purification and Analytical Characterization

The subsequent purification step involves cooling crystallization, a method predicated on forming a supersaturated solution that enables the preferential precipitation of curcumin crystals, with impurities persisting in the mother liquor. Sophisticated chromatographic methodologies represent the ultimate step in the purification and characterization processes, employing composite resins, including those formulated from polyamide-6 and poly(styrene-divinylbenzene), for the isolation of highly pure fractions of curcumin, demethoxycurcumin (DMC), and bisdemethoxycurcumin (BDMC) [31]. Analytical separation is primarily accomplished via High-Performance Liquid Chromatography (HPLC) with C18 stationary phases and ultraviolet detection, whereas advanced techniques such as High-Performance Countercurrent Chromatography (HPCCC) can achieve purities up to 99.5% [32].

## 2. Chemical Characterization: Synthetic and Biosynthetic Pathways

The chemical profile of curcumin is established through its synthesis and natural formation, irrespective of botanical extraction methods [33]. These complementary processes enable both the large-scale manufacturing of consistent curcuminoids and insight into the plant’s secondary metabolic pathways [33,34].

### 2.1. Total Chemical Synthesis

The Pabon method, predicated on an aldol condensation between vanillin (the aromatic aldehyde) and acetylacetone (the β-diketone), represents the most effective approach to the total synthesis of curcumin [35]; this process is precisely controlled using boron-based catalysts, including B_2_O_3_ or trialkyl borates, and tertiary amines that function to stabilize enolic intermediates and inhibit undesirable side reactions [35,36]. The borate system serves as a protective agent for the central diketone moiety, thereby preventing the formation of by-products [35]. Concurrently, trialkyl borates function as dehydrating agents, shifting the chemical equilibrium towards the formation of the desired heptadienedione conjugated system. This optimization generally yields synthetic products in the range of 60–80% [35,37].

### 2.2. Structural Analogues and Metal Complexes

Studies focusing on structural modifications have resulted in the development of diverse analogues aimed at mitigating the parent molecule’s intrinsic pharmacological deficiencies [38,39]. The advancements include the synthesis of monocarbonyl analogs of curcumin (MACs), which have demonstrated heightened chemical stability and more effective antimicrobial properties [40]. Moreover, it has been established that the complexation of curcumin with divalent metal ions, including Fe^2+^, Zn^2+^, and Mn^2+^, leads to a significant increase in the molecule’s oxidative degradation resistance and improved redox stability [41]. Curcumin’s beneficial potential is shifting from isolated nutraceutical interventions to comprehensive molecular combinations aimed at simultaneous modulation of multiple biological pathways. A key strategy involves the co-administration of curcumin with piperine, an alkaloid derived from black pepper. Piperine has been investigated as a bioenhancer because of its ability to inhibit intestinal and hepatic glucuronidation, thereby increasing the systemic exposure of curcumin. In a pharmacokinetic study in healthy volunteers, co-administration of piperine increased the relative bioavailability of curcumin by 2000% compared with curcumin administered alone [42]. However, enhanced bioavailability should not necessarily be considered an unequivocal pharmacological benefit, as piperine may also modulate drug-metabolizing enzymes and transporters involved in the disposition of concomitantly administered compounds. In particular, inhibition of CYP3A4 and P-glycoprotein has been reported, raising the possibility of altered plasma concentrations and pharmacokinetic profiles of co-administered medications. Therefore, the use of piperine as a bioenhancer should be considered in the context of its potential for drug–supplement interactions, particularly when administered together with drugs with a narrow therapeutic index [43].

In addition to metabolic enhancers, the formation of stable coordination complexes through physical complexation with divalent transition metal ions like zinc (Zn^2+^), iron (Fe^2+^), and manganese (Mn^2+^) represents a significant approach. This strategy effectively immobilizes the central dione moiety of curcumin, thereby mitigating hydrolytic degradation and augmenting its capacity to scavenge localized free radical cascades [41]. Moreover, the potential use of curcumin in combination with established neuropharmacological agents has been investigated in experimental settings, including combinations with standardized Ginkgo biloba extract in Alzheimer’s disease (AD) models and with levodopa in Parkinson’s disease (PD) models. These experimental studies suggest that curcumin may exert complementary neuroprotective effects, including attenuation of oxidative stress and modulation of dopaminergic and cholinergic neurotransmission [44,45].

### 2.3. Biosynthesis in Curcuma longa

Natural biosynthesis proceeds via the phenylpropanoid pathway. Phenylalanine Ammonia-Lyase (PAL) initiates this process, converting phenylalanine to cinnamic acid, which is subsequently hydroxylated and methylated to produce ferulic acid and other essential aromatic precursors [46]. These intermediates are subsequently activated into CoA thioesters by 4-coumarate: CoA ligase (4CL), and these thioesters then act as substrates for Type III polyketide synthases. The specific profiles of curcumin, DMC, and BDMC are achieved through the orchestrated condensation of these precursors by Diketide-CoA Synthase (DCS) and various isoforms of Curcumin Synthase (CURS1, CURS2, and CURS3) [47,48,49]. The more streamlined biosynthetic route is also offered by the single-enzyme mechanism of Curcuminoid Synthase (CUS) [49,50].

## 3. General Properties: Molecular Architecture and Pharmacokinetics

### 3.1. Structural Pharmacology

Curcumin, with the chemical designation (1E,6E)-1,7-bis(4-hydroxy-3-methoxyphenyl)-1,6-heptadiene-3,5-dione (MW 368.38), is a symmetrical compound featuring two methoxyphenolic rings connected by an unsaturated seven-carbon linker that includes a β-diketone moiety [36]. The structural characteristics, which were extensively employed in ancient medical systems as recorded in the Sushruta Samhita and Charaka Samhita, are the chief contributor to its varied biological actions [51].

### 3.2. Molecular Targets

Curcumin exhibits pleiotropic regulatory activity on signaling cascades at the cellular level, characterized by its effective inhibition of the pro-inflammatory nuclear factor-kappa B (NF-κB) and modulation of the mitogen-activated protein (MAP) kinases and PI3K/Akt pathways [52,53,54]. The neuroprotective potential of this compound is significantly increased due to its ability to cross the blood–brain barrier (BBB) and impact epigenetic regulations like histone acetylation and DNA methylation, which play a crucial role in neurodegenerative disease progression [55,56,57].

### 3.3. Bioavailability Constraints and Delivery Systems

Although curcumin possesses a high therapeutic index, it is subject to a substantial “bioavailability paradox” [58]. This phenomenon is defined by severely limited intestinal absorption, swift hepatic conjugation (glucuronidation and sulfation), and rapid elimination from the systemic circulation [58,59]. According to preclinical studies, concentrations of 400 mg/kg diminish to undetectable levels in the bloodstream within two hours [60]. To address these metabolic limitations, scientific inquiry has pivoted towards novel delivery mechanisms, encompassing nanoparticles, liposomes, and phospholipid complexes, with the objective of increasing systemic persistence and augmenting therapeutic outcomes [61,62,63,64].

To overcome the “bioavailability paradox,” which is influenced by extensive phase II hepatic conjugation (glucuronidation and sulfation) and P-glycoprotein-mediated efflux, the new strategies has shifted towards the comparative biophysical engineering of sophisticated curcumin delivery platforms. Solid lipid nanoparticles (SLNs), such as the Longvida formulation, employ a lipid-bilayer structure that enhances the absorption of lipophilic substances through cell membranes and aids in lymphatic delivery, consequently bypassing initial liver metabolism and resulting in elevated levels of free curcumin in brain tissue [65]. Conversely, submicron crystal solid dispersions, exemplified by Theracurmin, leverage colloidal suspension technologies to significantly diminish particle size to the nanoscale, thereby improving the dissolution rate and gastrointestinal absorption profile [66]. Additionally, self-assembling hydrogel complexes, derived from natural polymers, exemplified by curcumin-galactomannan (CurQfen), utilize a hydrophilic mucilage matrix to shield the lipophilic dione core from degradation. This mechanism facilitates sustained systemic release and enhances BBB permeability [67]. Pharmacokinetic comparisons reveal that SLNs enhance prolonged tissue retention, whereas galactomannan complexes yield higher peak plasma levels of unbound curcumin, underscoring the need to adapt formulation matrices to particular kinetic requirements of the targeted neuropathology [61,63].

## 4. Curcumin, Cellular Homeostasis and Neuroinflammation: Mechanistic Insights

Curcumin is crucial for maintaining cellular homeostasis in the central nervous system, specifically by counteracting inflammation, mitochondrial dysfunction and the resultant overproduction of reactive oxygen species (ROS) (Figure 2) [68,69].

Mitochondrial health is supported by modulating mitophagy and the PI3K/Akt/mTOR signaling, which is vital for protecting neurons from apoptotic cell death and enabling synaptic plasticity [70,71]. The principal anti-inflammatory effect of curcumin is achieved through the inhibition of the NF-κB signaling cascade, thereby diminishing the synthesis of inflammatory cytokines including TNF-α, IL-1β, and IL-6, in addition to inflammatory enzymes such as COX-2 and iNOS [72,73]. The cessation of the self-amplifying inflammatory cycle is essential for mitigating the exacerbation of neurodegenerative diseases [74,75]. Curcumin exerts its powerful antioxidant effects through two primary mechanisms: directly neutralizing free radicals, including superoxide anions (O_2_^−^) and hydroxyl radicals (OH), via its phenolic hydroxyl groups [76,77] and indirectly activating the Nrf2-ARE pathway. The latter facilitates the movement of Nrf2 into the nucleus, thereby initiating the synchronized production of protective enzymes such as SOD, CAT, and HO-1, which collectively provide strong resistance to persistent oxidative stress [78,79].

### 4.1. Curcumin and Gut–Brain Axis

The gut–brain axis represents a bidirectional communication network linking the gastrointestinal tract, the intestinal microbiota, the enteric nervous system, and the central nervous system. Current evidence indicates that curcumin can interact with the gut microbiota and undergo microbial biotransformation, generating metabolites (primarily tetrahydrocurcumin (THC) and hexahydrocurcumin) that may differ from the parent compound in terms of stability, solubility, and biological activity [80,81]. These metabolites demonstrate improved water solubility, increased chemical resilience under physiological pH conditions, and an extended systemic duration of action relative to the original curcumin [82]. Concurrently, the local immunomodulatory influence of these metabolites results in enhanced expression of tight junction proteins such as occludin and zonula occludens-1 (ZO-1). This mechanism serves to preserve intestinal barrier integrity and obstruct the systemic translocation of neurotoxic lipopolysaccharides (LPS) [83].

Experimental studies have also shown that curcumin can influence intestinal barrier function and microbial composition, suggesting that the gastrointestinal tract may represent an important site of action for orally administered curcumin [83,84].

Preclinical evidence provides further support for an interaction between curcumin and the gut–brain axis. In animal models, curcumin supplementation has been associated with changes in gut microbial composition, short-chain fatty acid production, and immune responses. For example, in a murine model of myasthenia gravis, curcumin modulated the gut microbiota and short-chain fatty acids and influenced the Th17/Treg balance [85]. In transgenic mice with Alzheimer’s disease, bidirectional interactions between curcumin and the gut microbiota were also reported, supporting a reciprocal relationship between intestinal microbial metabolism and curcumin activity [86]. These findings provide experimental evidence that modulation of the intestinal microbiota may contribute to the biological effects of curcumin. A possible mechanistic link between intestinal changes and neuroprotection has been proposed through the production of microbial metabolites, particularly short-chain fatty acids (SCFAs) such as butyrate, as well as through the maintenance of intestinal barrier integrity and the regulation of systemic immune and inflammatory responses [81,82,83]. These mechanisms could, in turn, influence neuroinflammatory processes through gut–brain communication. However, the causal relationship between curcumin-induced microbiota alterations and specific neurological outcomes remains to be fully established, particularly in humans. Human evidence is currently more limited and largely indirect. A randomized controlled trial in obese individuals reported that curcumin supplementation increased the abundance of butyrogenic bacteria in the gut [87]. Although this finding supports the ability of curcumin to modulate the human gut microbiota, the study did not establish a direct relationship between these microbiota changes, gut–brain signaling, and neurological outcomes. Similarly, clinical trials reporting cognitive effects of bioavailable curcumin formulations have not directly demonstrated that these effects are mediated by changes in the gut microbiota [65,66,67]. Therefore, the gut–brain axis should currently be regarded as a plausible contributor to the neurobiological effects of curcumin, supported by mechanistic and preclinical evidence but not yet conclusively demonstrated as a causal pathway in humans.

### 4.2. Curcumin’s Role as a Neuromodulator in M1/M2 Microglial Polarization

Curcumin operates as a promising neuromodulator by facilitating the shift in microglial phenotypes from the pro-inflammatory M1 to the anti-inflammatory M2 state (Figure 3).

While the M1/M2 classification has offered a valuable conceptual framework for characterizing specific activation programs in experimental models of microglia and macrophages, recent insights into microglial biology have revealed significant limitations of this binary model [74,75]. Particularly, the classification of the M1 phenotype as pro-inflammatory and potentially neurotoxic, alongside the M2 phenotype as anti-inflammatory, neuroprotective, and reparative, tends to characterise the microglial response as a set of distinct and mutually exclusive states. Nonetheless, microglia possess high plasticity and can modulate several molecular and functional processes simultaneously, depending on the biological context [74]. The M1/M2 categorization is chiefly based on experimental investigations first carried out in macrophages and subsequently utilised to characterise microglia, involving specific immune stimuli such as LPS, IFN-γ, or IL-4 to elicit relatively defined activation pathways. However, these experimental settings do not entirely mimic the intricate in vivo central nervous system microenvironment, in which microglia encounter multiple cellular and molecular stimuli simultaneously [88,89]. Microglia have been historically characterised in scientific literature by a binary framework, correlating M1 programmes with pro-inflammatory markers such as iNOS/NOS2, CD86, MHC-II, IL-1β, TNF-α, and IL-6, and M2 programmes with markers like CD206/MRC1, Arg1, Ym1/2, IL-10, and TGF-β [74,75].

In this context, curcumin may play a fundamental role in reestablishing immunological balance, reducing chronic neuroinflammation, and promoting neuroprotection primarily through TLR4/NF-kappaB inhibition and PPAR-gamma activation [72,73]. Nevertheless, its translation to clinical and in vivo settings is markedly limited by critical pharmacokinetic barriers [58,59]. They include significantly low aqueous solubility, rapid first-pass metabolism, chemical instability under physiological pH, and inadequate blood–brain barrier penetration, resulting in free brain concentrations at nanomolar levels [37,58]. Therefore, these levels are insufficient to meet the micromolar thresholds required in vitro, with higher doses causing cellular toxicity [68].

## 5. In Vitro Evidence for Curcumin

In vitro investigations, predominantly with BV2microglial and U87 neuroglioma cell lines, have yielded significant understanding of curcumin’s immunomodulatory functions. In these contexts, curcumin serves to effectively downregulate the TLR4/MyD88/NF-κB signaling pathway and inhibit the NLRP3 inflammasome, leading to a pronounced reduction in IL-1β and other neurotoxic factor secretion (Table 1). Microglial activation and the subsequent chain of neuroinflammatory occurrences are significant pathological hallmarks observed in a variety of central nervous system disorders, encompassing acute traumatic injuries, chronic neurodegenerative conditions, and oncological malignancies. On the cell surface, the Toll-like receptor 4 (TLR4) complex functions as a key component in the initiation of these inflammatory pathways. The findings of Youn et al. (2006) indicate that inhibiting the dimerization of the TLR4 complex is a successful strategy for blocking the downstream activation of both MyD88-dependent NF-κB and TRIF-dependent IRF3 [90]. Zhu et al. (2014) further emphasize the potential protective significance of regulating this receptor cascade in acute pathological states, demonstrating that blocking TLR4/MyD88/NF-κB signaling provides substantial neuroprotection and anti-inflammatory benefits, subsequently to traumatic brain injury [91]. The implicated signaling axis extends beyond physical trauma to tumor microenvironments, as detailed by Bi et al. (2021) [92]. Their research indicates that inhibiting the HSP60/TLR4/MYD88/NF-κB pathway effectively curtails proliferation and promotes apoptosis in U87 glioma cells [92]. The regulation of downstream transcriptional responses and the synthesis of inflammatory mediators is meticulously controlled by the MAPK (Mitogen-activated protein kinase) and NF-κB pathways in response to different exogenous and endogenous stimuli. For example, Gu Kang et al. (2004) reported that curcumin successfully attenuates cyclooxygenase-2 (COX-2) expression in LPS-stimulated BV2 microglial cells by simultaneously blocking the NF-κB and AP-1 signaling pathways [93]. In a comparable study, Yangyang Yu et al. (2018) demonstrated that inhibiting MAPK/NF-κB signaling in lipoteichoic acid (LTA)-induced BV-2 microglia substantially reduces the generation of pro-inflammatory mediators [94]. Moreover, the reaction to bacterial elements like Pam3CSK4 is subject to modification; Meiling Jin and colleagues (2017) demonstrated a strong correlation between the attenuation of pro-inflammatory mediators in Pam3CSK4-stimulated BV-2 cells and the activation of the cytoprotective Nrf2/HO-1 pathway [95]. The NLRP3 inflammasome contributes distinctly but harmoniously to the maintenance of neuroinflammation by serving as a principal platform for the maturation of crucial cytokines. Qian Shi and colleagues (2021) established that diminishing the NLRP3 inflammasome pathway serves to restrict microglial activation and the ensuing generation of interleukin-1β (IL-1β) within BV-2 cells [96]. Enzymatic cleavage plays a pivotal role in this cascade’s mechanism, with research by Haipeng Yin et al. (2018) showing that direct inhibition of caspase-1 activity prevents NLRP3 activation and blocks IL-1β secretion, thereby establishing caspase-1 as a critical molecular target for anti-inflammatory interventions [97]. Current efforts are increasingly aimed at modulating the functional characteristics of microglia to promote neuroprotection. According to Zhang et al. (2019), enhancing polarization toward the M2 microglial phenotype demonstrably reduces the production of pro-inflammatory cytokines IL-1β and IL-6, thereby ameliorating neuroinflammation [98]. Complex intracellular signaling networks direct this phenotypic transition. In this context, research by Chiara Porro et al. (2019) revealed that the inhibition of the JAK2/STAT3 signaling cascade within microglia enhances the expression of the regulatory anti-inflammatory cytokines IL-4 and IL-10 [99]. Ultimately, protecting glial cells from environmental and chemical harm is a necessary condition for the maintenance of cellular balance. Parada and colleagues (2015) assessed the protective capabilities of glial cells under combined oxidative and inflammatory stress conditions. The research demonstrated that enhancing an anti-inflammatory microglial phenotype through a heme oxygenase-1 (HO-1)-dependent pathway effectively reduces the expression of inducible nitric oxide synthase (iNOS) [100]. Regarding heavy metal-induced neurotoxicity, particularly in instances of Mn^2+^ exposure, Euteum Park and colleagues (2017) established that apoptosis resulting from Mn^2+^ exposure in BV-2 microglial cells may be circumvented through modifications to the equilibrium of apoptotic regulators. This involves a reduction in the expression of the pro-apoptotic protein Bax and caspases, coupled with an elevation in the levels of the anti-apoptotic protein Bcl-2 [101].

Curcumin is demonstrating potential as a key immunomodulator in neuro-oncology, capable of interfering with the pathological interplay within the glioblastoma multiforme (GBM) tumor microenvironment, specifically by targeting glioma-associated microglia/macrophages (GAMs). The development of an immunosuppressive, tumor-promoting M2-like phenotype in infiltrating myeloid cells is actively manipulated by high-grade gliomas, thereby enabling immune evasion and tissue invasion. Curcumin administration successfully inhibits the HSP60/TLR4/MyD88/NF-κB signaling pathway in U87 glioma cells and surrounding glioma-associated macrophages, producing a dual benefit [92]. The compound promotes glioma stem cell apoptosis and cell-cycle arrest, while also inducing phenotypic repolarization of glioma-associated macrophages from an M2 (tumor-supportive) to an M1 (pro-inflammatory, tumor-destructive) state [98]. The microenvironment’s alteration results in a significant reduction in the secretion of immunosuppressive cytokines, specifically transforming growth factor-beta (TGF-β) and interleukin-10 (IL-10). Simultaneously, there is an augmentation of tumor-necrosis factor-alpha (TNF-α) and nitric oxide synthase. Consequently, host immune surveillance mechanisms are restored, thereby enhancing the synergistic effectiveness of standard chemotherapeutics, like temozolomide [57,99].

## 6. In Vivo Evidence for Curcumin

Animal models have consistently demonstrated the capacity of curcumin to preserve the structural integrity of the BBB and modulate the neuroimmune environment. In models ranging from neonatal white matter injury to adult Parkinson’s and ischemic stroke, curcumin treatment consistently favors the polarization of microglia toward the neuroprotective M2 phenotype, reducing the levels of pro-inflammatory enzymes and enhancing functional outcomes (Table 2).

The neuroprotective potential of curcumin has been corroborated by numerous in vivo studies, which demonstrate its efficacy in modulating microglial activation and neuroinflammatory processes relevant to neurodegeneration. The intraperitoneal administration of curcumin (50 mg/kg) to neonatal Sprague-Dawley rats was observed to significantly alleviate white matter injury and pre-oligodendrocyte mortality, concurrently with a downregulation of iNOS and NOX subunits (p67phox and gp91phox) in microglia [102]. In a parallel finding, C57BL/6J mice treated with curcumin loaded into exosomes exhibited a reduction in brain inflammation within an experimental autoimmune encephalomyelitis model, accompanied by induced microglial apoptosis [103]. The neuroprotective properties of nano-curcumin have been documented in animal models of subarachnoid hemorrhage. In these studies, curcumin encapsulated within poly (lactic-co-glycolic acid) (PLGA) nanoparticles effectively maintained the integrity of the BBB (Figure 4).

This was achieved through the inhibition of tight junction protein disruption, as well as a reduction in oxidative stress, inflammatory reactions, and programmed cell death [104]. Concurrently, in murine models of PD induced by 6-hydroxydopamine (6-OHDA), curcumin demonstrated a neuroprotective effect on nigrostriatal dopaminergic neurons, leading to a reduction in astrocytic and microglial activation [105].

It has been shown that curcumin possesses the ability to directly influence microglial reactions. In murine models of ischemic stroke, the application of curcumin promoted microglial polarization to the M2 phenotype, consequently yielding diminished inflammatory responses, attenuating neuronal harm, and improved functional outcomes [106]. Likewise, within experimental paradigms of traumatic brain injury, intraperitoneal delivery of curcumin has been observed to diminish the prevalence of TLR4-positive microglia/macrophages. This intervention also led to a reduction in the secretion of pro-inflammatory cytokines, such as IL-1β, TNF-α, and MCP-1, via the suppression of the TLR4/MyD88/NF-κB signaling cascade [91]. Further experimental evidence has now reinforced the significance of curcumin in controlling neuroinflammation and microglial polarization. Functional recovery and modulation of the neuroinflammatory response were observed in a murine model of spinal cord injury following the transplantation of curcumin-activated olfactory ensheathing cells (aOECs). This improvement is attributed to the promotion of microglial polarization towards an M2-like phenotype, mediated by the APOE/TREM2/NF-kB pathway [107]. Analogously, in studies involving spinal cord ischemia–reperfusion injury models, curcumin treatment effectively reduced oxidative stress and neuroinflammation by modulating the Nrf2/NF-κB signaling pathway [108].

Additional in vivo research has indicated that the transplantation of aOECs has the capacity to reduce traumatic neuronal damage and accelerate functional recovery after spinal cord injury. This is accomplished through the regulation of inflammatory pathways and the promotion of M2 polarization in microglia/macrophages, mediated by olfactory glial-derived interleukin-4 (IL-4) [109]. Emerging data suggest that Curcuma’s bioactive constituents, exemplified by curcumol, can effectively reduce neuroinflammation in cerebral ischemia–reperfusion models via the regulation of microglial polarization and the Nrf2/HO-1 and NF-κB pathways [110]. To summarize, the evidence corroborates the protective promise of curcumin for neurodegenerative and neuroimmune disorders distinguished by persistent neuroinflammation, chronic microglial activation, and oxidative stress.

## 7. Clinical Evidence for Curcumin

Human clinical trials have investigated the neuroprotective potential of various bioavailable curcumin formulations, including Theracurmin, Longvida, CurQfen, and Curcumin C3 Complex^®^. While significant improvements in cognitive performance and reduced cerebral amyloid/tau burden have been observed in healthy elderly populations, results in symptomatic Alzheimer’s and Parkinson’s patients remain heterogeneous due to methodological differences and bioavailability limitations (Table 3).

The advancement of curcumin for human neurological treatments, originating from preclinical studies, has been largely defined by endeavors to enhance its systemic availability. The main focus has been on engineering sophisticated delivery vehicles such as liposomes, micelles, and nanoparticles. These advanced formulations have proven capable of traversing the BBB and conferring neuroprotective benefits in healthy, non-demented older adults, thereby impacting cognitive aging and biomarker accumulation. A randomized, double-blind, placebo-controlled trial lasting 18 months was conducted by Gary W. Small and colleagues (2018) to assess the efficacy of Theracurmin, a bioavailable submicron crystal solid dispersion of curcumin, administered at 90 mg twice daily to 40 non-demented adults aged 51 to 84 [66]. The findings of this study revealed that patients receiving Theracurmin orally on a daily basis experienced substantial improvements in both verbal memory, evaluated using the Buschke Selective Reminding Test (SRT), and visual memory, alongside enhanced attention as assessed by the Trail Making Test A. Crucially, longitudinal FDDNP-PET imaging demonstrated that the observed cognitive improvements correlated with a significant reduction in amyloid-β and tau tracer uptake within the amygdala and hypothalamus. These brain regions are integral to mood and memory regulation, thus offering direct in vivo evidence of plaque and tangle modulation in the human brain [66].

Alongside the clinical trial, a bioavailability study was conducted evaluating the Theracurmin formulation against GNC Herbal Plus Standardised Turmeric^®^ (supplied by GNC [Pittsburgh, PA, USA]) and Meriva^®^ (supplied by Indena SpA [Milan, Italy]). To evaluate the plasma curcumin bioavailability of Theracurmin^®^, an assessment was conducted measuring the levels following the administration of a single dose of three Theracurmin^®^ capsules (each containing 31 mg of curcumin), two Meriva^®^ capsules (each 51 mg), and one GNC Herbal Plus^®^ Standardised Turmeric capsule (433 mg). Researchers assessed the kinetics of curcumin absorption by collecting blood samples at 0.5, 1, 2, 4, and 6 h after the participants ingested the capsules. The bioavailability study involved comparing three types of curcumin in 10 healthy volunteers. Theracurmin^®^ demonstrated a more rapid absorption (T-max: 2 h) relative to GNC Herbal Plus Standardised Turmeric^®^ and Meriva^®^. Administration of Theracurmin resulted in significantly elevated peak concentrations (40.1 ± 13.3 µg/L) and areas under the curve (165 ± 64 µg × hour/L) in comparison to GNC Herbal Plus Turmeric (23.4 ± 18.8) [66].

This pilot study has several limitations. First, the small sample size limits the generalizability of the findings and precludes subgroup analyses regarding the impact of Mild Cognitive Impairment (MCI). Second, given the exploratory nature of the trial, corrections for multiple comparisons were not applied, and FDDNP-PET imaging was restricted to a subset of the cohort. Finally, although educational attainment was statistically controlled for, potential residual confounding from minor baseline differences between treatment groups cannot be completely ruled out [66].

Katherine H. M. Cox et al. (2015) conducted a study that explored the immediate and sustained effects of a specific curcumin formulation, Longvida, on cognitive function, mood, and blood parameters in healthy adults aged 50 to 80 [65]. This research, which involved administering 400 mg of Longvida daily (comprising 80 mg of free curcumin in a solid lipid formulation, remaining weight comprised commonly used pharmaceutical excipients and small amounts of other curcuminoids present in turmeric extract), complements existing long-term findings [65].

This research, which involved administering 400 mg of Longvida daily (comprising 80 mg of free curcumin), complements existing long-term findings [65].

Findings from the double-blind, placebo-controlled assessment demonstrated that a single acute administration of Longvida substantially augmented working memory function and sustained attention from one to three hours post-ingestion. Following a 28-day period of chronic supplementation, participants demonstrated sustained enhancements in both working memory and spatial memory. Additionally, notable decreases in fatigue, anxiety, and stress reactivity were observed, thereby validating the efficacy of bioavailable curcumin in promoting healthy cognitive aging and psychological resilience [65]. This clinical trial presents several limitations. First, the relatively small sample size (N = 60) comprised exclusively of healthy older adults, which constrains the generalizability of the findings to populations with cognitive impairment or neurodegenerative disorders. Furthermore, the 4-week intervention period was insufficient to establish the long-term efficacy and safety profile of the solid lipid curcumin formulation [65].

Conversely, clinical investigations focused on established AD pathology reveal a distinct evolutionary progression, moving from conventional herbal treatments to sophisticated, bioenhanced molecular compounds. Nozomi Hishikawa et al. (2012) presented a case study on the therapeutic effects of Ayurvedic medicine, administering turmeric powder capsules to three patients diagnosed with severe AD and significant behavioral and psychological symptoms of dementia (BPSD), including pronounced irritability, agitation, anxiety, apathy, and wandering [111]. The administration of turmeric therapy resulted in considerable behavioral improvements in patients, including significant mitigation of neuropsychiatric symptoms, a decrease in urinary incontinence, and some positive trends in general cognitive functioning [111]. However, the process of formalizing these discoveries within comprehensive randomized controlled trials (RCTs) employing unformulated curcumin presented considerable obstacles, primarily attributable to insufficient absorption. Larry Baum and colleagues (2008) executed a 6-month, double-blind, randomized pilot investigation involving 34 AD patients. This study contrasted daily oral administration of 1 g or 4 g of standard curcumin, alongside 120 mg of standardized ginkgo leaf extract for all participants, with a placebo [44]. Although the intervention demonstrated safety and high tolerability, it did not yield statistically significant differences in Mini-Mental State Examination (MMSE) scores between the groups. Nevertheless, preliminary analyses of serum samples indicated potential patterns in amyloid-β aggregation and systemic oxidative biomarkers [44]. Further characterization of this therapeutic plateau was provided by John M. Ringman et al. (2012) through a 24-week, double-blind, placebo-controlled study of Curcumin C3 Complex (up to 4 g daily) conducted on 36 patients diagnosed with mild-to-moderate AD [112]. A pharmacokinetic analysis was also carried out to assess the levels of curcumin and its metabolites in blood sample. HPLC analysis revealed that native curcumin was undetectable in all post-dose samples, except for a single sample collected 4 h after administration of a 4 g dose, in which a concentration of 6 ng/mL was detected. Consequently, the more sensitive LC/MS/MS method was employed to quantify native curcumin, free tetrahydrocurcumin, and their respective glucuronidated metabolites [112].

At the 24-week visit, mean baseline plasma concentrations of native curcumin and free tetrahydrocurcumin were 2.67 ± 1.69 ng/mL and 6.87 ± 4.91 ng/mL, respectively (<10% CV). Three hours following drug administration, mean plasma concentrations were 7.76 ± 3.23 ng/mL for native curcumin and 3.73 ± 2.0 ng/mL for tetrahydrocurcumin. The corresponding concentrations of glucuronidated curcumin and glucuronidated tetrahydrocurcumin were 96.05 ± 26 ng/mL and 298.2 ± 140.04 ng/mL, respectively.

Plasma curcumin was undetectable in placebo-treated subjects and in samples collected prior to curcumin administration. Similarly, native curcumin was not detectable in cerebrospinal fluid (CSF) from either placebo-treated or curcumin-treated subjects [112].

Although the trial confirmed excellent safety and tolerability, it did not demonstrate significant clinical benefits on primary cognitive and behavioral scales such as the ADAS-cog, NPI, and ADCS-ADL. Furthermore, no measurable changes in plasma or cerebrospinal fluid biomarkers of AD pathology were induced [112]. Given the recognition of these structural impediments, recent scholarly efforts have been directed towards innovative delivery mechanisms. In 2023, S. Syam Das et al. conducted an evaluation of CurQfen, a highly bioavailable curcumin-galactomannan formulation developed to optimize BBB permeability [67]. A randomized controlled trial involving 48 individuals with mild cognitive impairment due to AD demonstrated that daily administration of 400 mg of CurQfen, taken twice daily, led to significant enhancements in executive cognitive function and neurocognitive assessments when contrasted with a placebo and unformulated curcumin. Unformulated standard curcumin (>95% purity, 10.8% curcumin content) was obtained from dried Cambodian turmeric (*Curcuma longa* L.) via ethyl acetate extraction. The test product, CGM, is a water-dispersible, proprietary formulation of USC combined with fenugreek galactomannan fibre. Identical bottles contained 65 sachets each of USC, MCC (placebo) or CGM. Each sachet, weighing approximately 3 g, contained 400 mg of the respective test material alongside maltodextrin (<10 dextrose equivalents). Furthermore, this supplementation favorably altered blood–brain barrier-permeable biomarkers associated with neurodegeneration, encompassing amyloid-β, tau, BDNF, and subsequent neuroinflammatory indicators [67]. Several methodological limitations of this study should be acknowledged: the cognitive evaluation relied solely on a single screening instrument (the MMSE), and the diagnostic framework lacked the ATN (Amyloid/Tau/Neurodegeneration) classification system. Finally, the absence of plasma curcumin concentration measurements precludes establishing direct pharmacokinetic-pharmacodynamic correlations [67].

Beyond its investigation in other neurodegenerative proteinopathies, curcumin’s therapeutic value has been assessed in PD. In PD, the progressive depletion of dopaminergic cells is precipitated by alpha-synuclein buildup and impaired mitochondrial function. A 9-month, triple-blind, randomized, placebo-controlled study by Hamidreza Ghodsi et al. (2022) assessed the efficacy of nanomicellar curcumin (80 mg/day) as an adjunctive therapy in 60 patients diagnosed with idiopathic PD [113].

Utilizing the MDS-UPDRS and PDQ-39, the study concluded that the nanomicellar formulation was entirely safe; however, it failed to produce statistically significant improvements in motor or non-motor trajectory or alter patients’ subjective quality of life relative to the placebo group [113]. The findings should be interpreted in light of several limitations. The small sample size limits the generalizability of the results, while heterogeneity in disease severity and duration may have obscured the potential neuroprotective effects of curcumin [113]. In contrast, Vincenzo Donadio and colleagues (2022) investigated the clinical and histological consequences of oral curcumin supplementation in individuals diagnosed with idiopathic PD, concentrating particularly on peripheral neuropathology [45]. Furthermore, a pharmacokinetic analysis of curcumin metabolites in plasma and cerebrospinal fluids revealed that patients who did not receive curcumin showed no detectable levels of curcumin, curcuminoids, or their metabolites in either plasma or cerebrospinal fluid (CSF), supporting the reliability of the analytical method. After 12 months of curcumin supplementation, plasma concentrations measured before and after the final morning dose showed a significant increase in total curcumin, DMC (*p* < 0.05), and BDMC (*p* < 0.05), whereas THC and HHC levels remained unchanged. Plasma THC and HHC reached steady-state concentrations. Notably, HHC and THC were detectable in the CSF of supplemented patients, although at lower concentrations than in plasma, while total curcumin, DMC, and BDMC remained undetectable in CSF. This clinical investigation demonstrated that curcumin supplementation yielded a quantifiable amelioration of non-motor clinical manifestations, alongside a notable decrease in the pathological aggregation of phosphorylated α-synuclein within cutaneous autonomic nerves, thereby offering a potential histopathological indicator of curcumin’s systemic disease-modifying efficacy [45]. The relatively small sample size and non-randomized design warrant confirmation in larger, double-blind, controlled trials. In addition, dietary and lifestyle factors potentially affecting levodopa responsiveness were not systematically monitored, although patients reported no relevant changes during follow-up [45]. In addition to chronic degenerative conditions, curcumin’s anti-inflammatory and vascular stabilizing attributes have demonstrated potential in neurovascular recovery after acute events, specifically during the rehabilitation period, subsequently to an ischemic stroke. A 12-week randomized, double-blind, placebo-controlled clinical trial by Kosar Boshagh et al. (2023) assessed the impact of co-supplementing curcumin with piperine in post-stroke rehabilitation patients [43]. The clinical trial indicated that the administration of curcumin-piperine daily resulted in a substantial decrease in systemic inflammatory indicators, notably high-sensitivity C-reactive protein (hs-CRP), and effectively alleviated systemic oxidative stress through an enhancement of Total Antioxidant Capacity (TAC). Moreover, the therapeutic intervention yielded positive modifications in cardiometabolic indices, characterized by substantial reductions in total cholesterol, serum triglycerides, systolic and diastolic blood pressure, and carotid intima-media thickness (CIMT) relative to the placebo cohort. This establishes a powerful, comprehensive therapeutic strategy for secondary prevention and vascular recuperation in the context of post-stroke rehabilitation [43]. This clinical trial presents several limitations, such as the advanced age of most participants, combined with the problems caused by the COVID-19 pandemic, affected recruitment and sample size. Furthermore, the lack of long-term follow-up also precludes conclusions concerning the durability of the observed effects. Finally, ethical considerations prevented evaluation of curcumin independently of piperine. For these considerations, the current results provide preliminary evidence supporting further investigation of curcumin-piperine supplementation during post-stroke rehabilitation [43].

## 8. Adulteration and Potency Deficiencies in Commercial Curcumin Supplements

The scientific view of curcumin has notably shifted over recent years: previously seen mainly as a pigment in *Curcuma longa*, it has now been identified as a multifunctional bioactive substance. Global preclinical and clinical studies have demonstrated its significant anti-inflammatory, antioxidant, and antirheumatic effects, along with encouraging findings in oncology [114,115,116,117]. These pharmacological profiles have led to the acknowledgement of curcumin as a compound of significant interest for human health and have prompted key regulatory bodies, including the U.S. Food and Drug Administration (FDA) and the European Food Safety Authority (EFSA), to evaluate its safety for human consumption [118,119]. From both regulatory and commercial viewpoints, the dietary supplement industry, which includes products designed to enhance the diet by supplying micronutrients, amino acids, or botanical extracts, has experienced significant growth [120]. This expansion has been enabled by extensive distribution, comprehensive marketing approaches, and regulatory frameworks that are typically less rigorous than those applied to pharmaceutical products. Such a dynamic raises essential questions about product quality and traceability in the commercial market, given that differences in supply-chain controls and the absence of universally accepted analytical standards may result in significant quality variation, potentially endangering consumers through exposure to formulations with limited efficacy or safety concerns. A key issue in current scientific studies and the commercial market involves the conceptual conflation of the whole plant and its distinct bioactive constituents. The erroneous belief has resulted in unethical trade practices, notably the marketing of synthetic curcumin or non-conforming products inaccurately labelled as standardized natural extracts [121]. These concerns have garnered clinical focus as well: during May and June 2019, the Italian Ministry of Health identified upwards of 20 cases of acute non-viral cholestatic hepatitis correlated with consumption of turmeric-based dietary supplements, commonly combined with piperine [122,123,124].

Despite the investigations failing to detect chemical contaminants or residual solvents, health authorities stressed that a direct causal link to turmeric itself could not be confirmed, highlighting the necessity of clearly differentiating between the botanical material and its isolated components [125,126,127].

In accordance with the 2018 recommendations from the European Medicines Agency (EMA), warnings were implemented concerning the use of turmeric-containing products in patients with existing liver conditions. A further essential consideration is the common occurrence of adulteration, influenced by economic constraints and the gains derived from synthetic compound usage. The strong yellow pigmentation of turmeric could potentially obscure undeclared materials, from starches and substandard dyes to hazardous compounds such as lead chromate [128,129]. The significant costs linked to the purification of naturally derived compounds might motivate fraudulent actors to replace botanical extracts with petrochemically derived synthetic curcumin. These practices are especially challenging to identify due to the limitations of conventional chromatographic methods, including HPLC and HPTLC, which may prove inadequate when assessing complex or multicomponent formulations where signal overlap can obscure the absence of naturally occurring constituents like ar-turmerone [130,131]. In order to address the constraints of traditional quality-control methods, research has progressively concentrated on sophisticated and orthogonal analytical techniques, such as nuclear magnetic resonance (NMR) spectroscopy, mass spectrometry (MS), countercurrent separation, isotope-ratio mass spectrometry (IRSM), and carbon-14 analysis. This method is particularly effective in detecting fossil-derived raw materials, as these materials lack the inherent radiocarbon signature characteristic of contemporary biomass [132,133,134,135,136].

In this scenario, recent studies have demonstrated that several dietary supplements connected to clinically reported hepatotoxicity incidents contained synthetic curcumin labeled as a natural botanical extract. By employing an integrated method combining quantitative NMR (qNMR) spectroscopy and countercurrent separation, researchers successfully identified residual impurities derived from synthetic manufacturing processes. These compounds were indicated as potential causes of the hepatic adverse effects noted and drew attention to the constraints of standard analytical controls [137].

By employing a non-destructive technique, high-resolution investigation of the structural properties of commercial products is possible, allowing for the identification of diverse curcumin polymorphic forms that may influence the stability and dissolution behaviour of the active compound, while simultaneously offering a method to detect the illicit substitution of natural botanical extracts with synthetic derivatives [138]. The global distribution of turmeric adulteration exhibits significant variations. Among jurisdictions with stringent regulatory regimes and oversight mechanisms, namely Australia, Canada, the European Union, Japan, New Zealand, South Korea, and the United States, this adulteration is reported to occur less frequently. Alternatively, in various geographical areas, notably South Asia and South America, significant instances of fraud involving the addition of diluents and synthetic dyes, including corn starch, have been documented [139]. According to the estimates provided by the authors, the adulteration rates were 20.0% for turmeric products overall, 20.4% for turmeric offered as a spice, and 22.0% for dietary supplements [139].

Investigations into the accuracy of “natural” labelling statements and the authenticity of turmeric-based dietary supplements have utilised orthogonal analytical strategies involving high-performance liquid chromatography (HPLC) alongside carbon-14 dating. The findings demonstrated significant inconsistencies between label claims and the true composition of the products: merely four out of the fourteen commercial samples examined were verified as authentic based on their declared composition [140]. The global development of the nutraceutical sector has, at last, improved product distribution through large-scale e-commerce sites including Amazon and eBay, alongside online pharmaceutical outlets. These formulations are often promoted as natural remedies linked to alleged benefits. According to Nounou et al., this circumstance raises critical questions about product safety, conformity to labeling assertions, and the presence of unreported adulterants. Regulatory deficiencies in the United States, where the production, marketing, and labelling of these products are governed by a framework significantly different from that of pharmaceutical products, may enable the extensive distribution of preparations not subjected to the same pre-market regulations as medicines. The scientific literature particularly indicates that some of these formulations might be adulterated or fraudulently created through the addition of undisclosed pharmaceutical active substances, despite their marketing as safe and natural. These findings highlight the necessity of enhancing regulatory oversight and analytical quality-control mechanisms to bolster product authenticity, guarantee adherence to labeling standards, and safeguard public health [141].

## 9. Discussion

The translational progression of curcumin from a botanical bioactive to an established neuroprotective agent is characterised by a fundamental paradox: significant preclinical pleiotropy versus variable human clinical effectiveness [142,143,144]. Both in vitro and in vivo models demonstrate that curcumin inhibits canonical neuroinflammatory pathways by downregulating TLR4/MyD88/NF-κB signaling [91] and NLRP3 inflammasome formation [96,97], while enhancing the cytoprotective Nrf2/ARE antioxidant pathway [77] and promoting microglial M1-to-M2 phenotypic shift [106,107]. However, the clinical relevance of these preclinical observations is restricted due to substantial pharmacokinetic limitations. Phase II biotransformation occurring swiftly—mainly via hepatic UDP-glucuronosyltransferase (UGT)-mediated glucuronidation and sulfation—together with P-glycoprotein-mediated efflux and limited blood–brain barrier (BBB) penetration, lowers systemic free curcumin concentrations to sub-therapeutic levels [143], which explains the equivocal clinical findings in trials aimed at established Alzheimer’s and Parkinson’s disease [44,112,113].

To address this bioavailability discrepancy, pharmaceutical research has branched into two mechanistic paradigms. The first paradigm is based on biophysical engineering, using solid lipid nanoparticles (Longvida) [65], submicron crystal dispersions (Theracurmin) [66], and galactomannan hydrogel complexes (CurQfen) [67] to prevent hydrolytic degradation of the lipophilic dione core, avoid first-pass hepatic metabolism, and penetrate the blood–brain barrier to enhance bioavailability within the central nervous system. These bioenhanced platforms exhibit measurable clinical efficacy in cases of non-demented aging and mild cognitive impairment, yet they introduce kinetic variability dependent on formulation, thereby complicating regulatory standardization.

From both a comparative pharmacokinetic and mechanistic standpoint, these three platforms resolve the “bioavailability paradox” via complementary biophysical pathways. Theracurmin maximizes gastrointestinal dissolution rates through particle-size minimization [66], Longvida focuses on lymphatic system absorption to avoid Phase II hepatic clearance and ensure sustained CNS tissue retention [65] and CurQfen uses a hydrophilic polysaccharide matrix to maximize peak systemic exposure of unbound curcuminoids [67]. Moreover, whereas Theracurmin and Longvida show cognitive improvement and neuroimaging biomarker changes mainly in healthy or non-demented elderly groups [65,66], CurQfen has demonstrated clinical cognitive and biochemical effectiveness specifically in the context of mild cognitive impairment [67].

The second paradigm regards curcumin’s minimal systemic absorption as a peripheral mechanism engaged in the gut–brain axis [80,81]. Unabsorbed intestinal curcumin is metabolized by bacteria into pharmacologically active hydrogenated metabolites, including tetrahydrocurcumin (THC) [82], while promoting the growth of short-chain fatty acid (SCFA)-producing taxa such as Bifidobacterium and Lactobacillus [84,85]. These microbial SCFAs enhance the integrity of intestinal epithelial tight junctions (occludin, ZO-1), prevent systemic lipopolysaccharide (LPS) translocation [83] and cross the BBB to indirectly reduce microglial neuroinflammation [80,85,87].

Beyond the aspects of drug delivery and gut–brain mechanics, translational reproducibility heavily depends on the product’s composition, standardization, and quality control. The clinical literature often presents persistent ambiguity because of the conceptual conflation between crude botanical *Curcuma longa* extracts and isolated, highly purified curcuminoid fractions (curcumin, demethoxycurcumin [DMC], and bisdemethoxycurcumin [BDMC]) [4,121]. Achieving pharmaceutical-grade standardization—such as purification with High-Performance Countercurrent Chromatography (HPCCC) yielding purities up to 99.5% combined with cooling crystallization—is critical to eliminate uncharacterized plant residues, ensure consistent bioequivalent dosing, and ascribe therapeutic outcomes exclusively to the active ingredient [31,32,145].

Furthermore, specific molecular combinations and co-administrations play a crucial role in determining the safety and pharmacokinetic profiles of curcumin. Systemic curcumin bioavailability can be increased by up to 2000% through co-formulation with piperine, a black pepper alkaloid that suppresses hepatic and intestinal UGT-mediated glucuronidation [42,43,146]. Piperine’s simultaneous inhibition of cytochrome P450 enzymes (primarily CYP3A4) and P-glycoprotein transporters disrupts extensive xenobiotic processing, elevating the risk of drug–supplement interactions, especially in conjunction with pharmacotherapies that have narrow therapeutic indices [43,146]. Conversely, the physical complexation involving divalent transition metal ions (Zn^2+^, Fe^2+^, Mn^2+^) offers a distinctive non-enzymatic strategy in bioengineering. Through coordination with the central β-diketone moiety, metal complexation stabilizes the dione linker, protecting curcumin against hydrolytic and oxidative degradation, augmenting redox stability, and elevating the capacity for localized free-radical scavenging [41]. Ultimately, a significant obstacle to clinical application and public health safety arises from prevalent economically motivated adulteration (EMA) in the nutraceutical market, with non-compliance rates ranging from 20.0 to 22.0% [139,140]. Fraudulent activities involve adding starch diluents, contaminating products with toxic industrial pigments such as lead chromate, and substituting natural botanical extracts unlawfully with petrochemical-based synthetic curcumin [121,128,129,130].

The adulteration results in direct clinical outcomes: in 2019, over 20 cases of acute non-viral cholestatic hepatitis connected to turmeric dietary supplements, frequently with piperine, were documented by health authorities [122,123,124]. Further analytical studies using quantitative NMR (qNMR) demonstrated that multiple hepatotoxic supplements comprised unverified synthetic curcumin contaminated with toxic processing impurities instead of pure botanical extracts. Since standard chromatographic techniques (HPLC and HPTLC) fail to distinguish synthetic processing residues or natural biomass from fossil-derived alternatives owing to spectral signal interference, the obligatory use of orthogonal analytical standards; particularly qNMR spectroscopy, Isotope-Ratio Mass Spectrometry (IRMS), and Carbon-14 radiocarbon dating, which identifies fossil-derived materials lacking current radiocarbon signatures, is necessary [135,136,137,138,139]. This is an essential requirement to ensure chemical authenticity, prevent liver toxicity, confirm bioequivalence, and protect consumer safety.

## 10. Conclusions and Future Perspectives

To summarise, curcumin constitutes a potent multitarget bioactive agent with significant neuroprotective potential, attributed to its wide-ranging anti-inflammatory, antioxidant, and neuroprotective activities evidenced in preclinical models. However, achieving clinical outcomes requires addressing essential pharmacokinetic limitations, particularly rapid metabolic degradation and poor central nervous system accessibility. Progress in biophysical delivery engineering and enhanced comprehension of gut–brain axis interactions provide promising pathways to achieve physiologically significant exposure. To establish curcumin as a validated neuroprotective agent, it is essential to implement rigorous standardization, carefully consider potential drug interactions with bioenhancers, and enforce strict quality control to prevent commercial adulteration and maintain product safety, authenticity, and clinical efficacy.

## Figures and Tables

**Figure 1:**
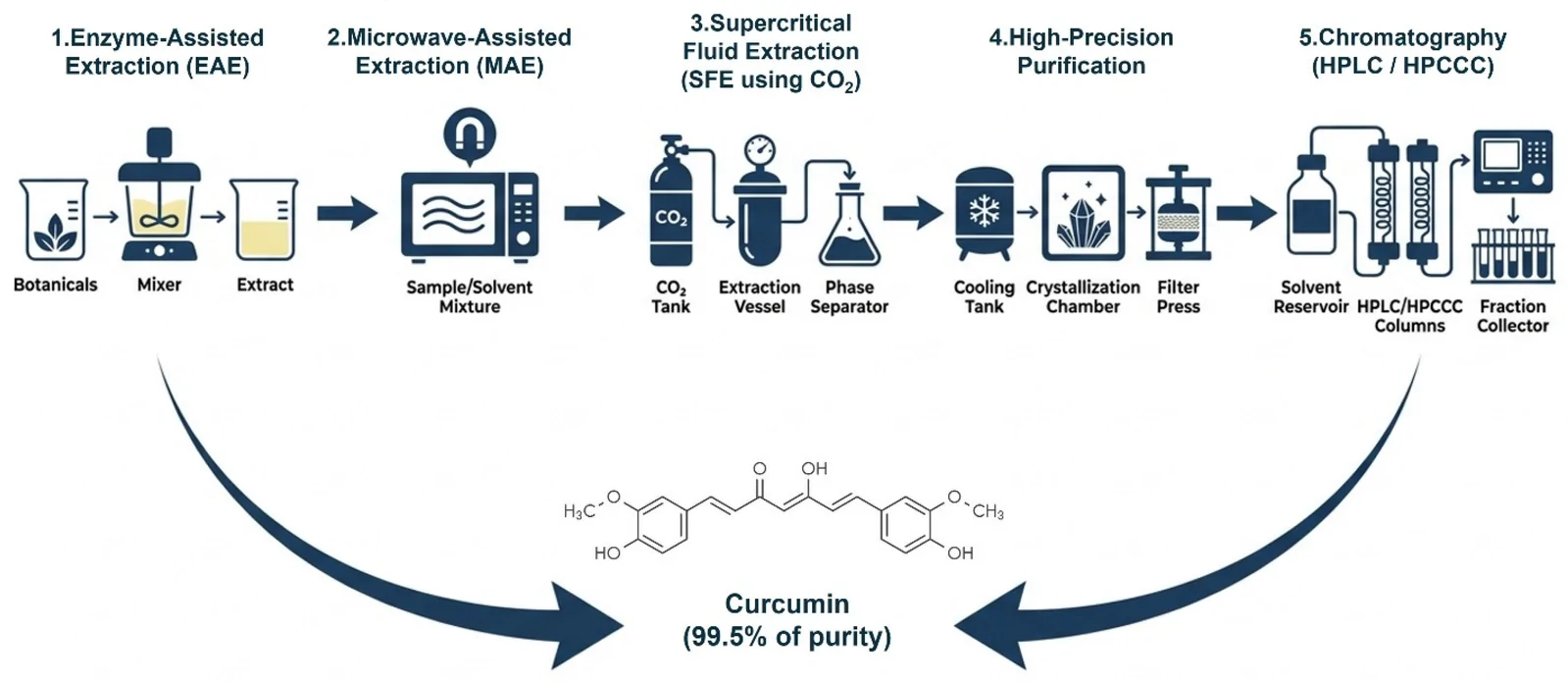
**Integrated green extraction and high-precision purification workflow of curcumin.** This flowchart illustrates the systematic process for isolating curcumin from botanical sources while maintaining high efficiency and sustainability. The process begins with green extraction techniques, specifically EAE, MAE, and SFE using CO_2_, which are employed to obtain a crude extract. This is followed by a high-precision purification phase involving cooling crystallization and advanced chromatography—such as HPLC H. PCCC. The final output of this integrated workflow is a 99.5% purity level, ensuring an optimal isolation of the active principle for advanced pharmacological applications and clinical trials. Abbreviations: Enzyme-Assisted Extraction (EAE); Microwave-Assisted Extraction (MAE); Supercritical Fluid Extraction (SFE); High-Precision Purification (HPP); High-performance liquid chromatography (HPLC); high-performance countercurrent chromatography (HPCCC).

**Figure 2:**
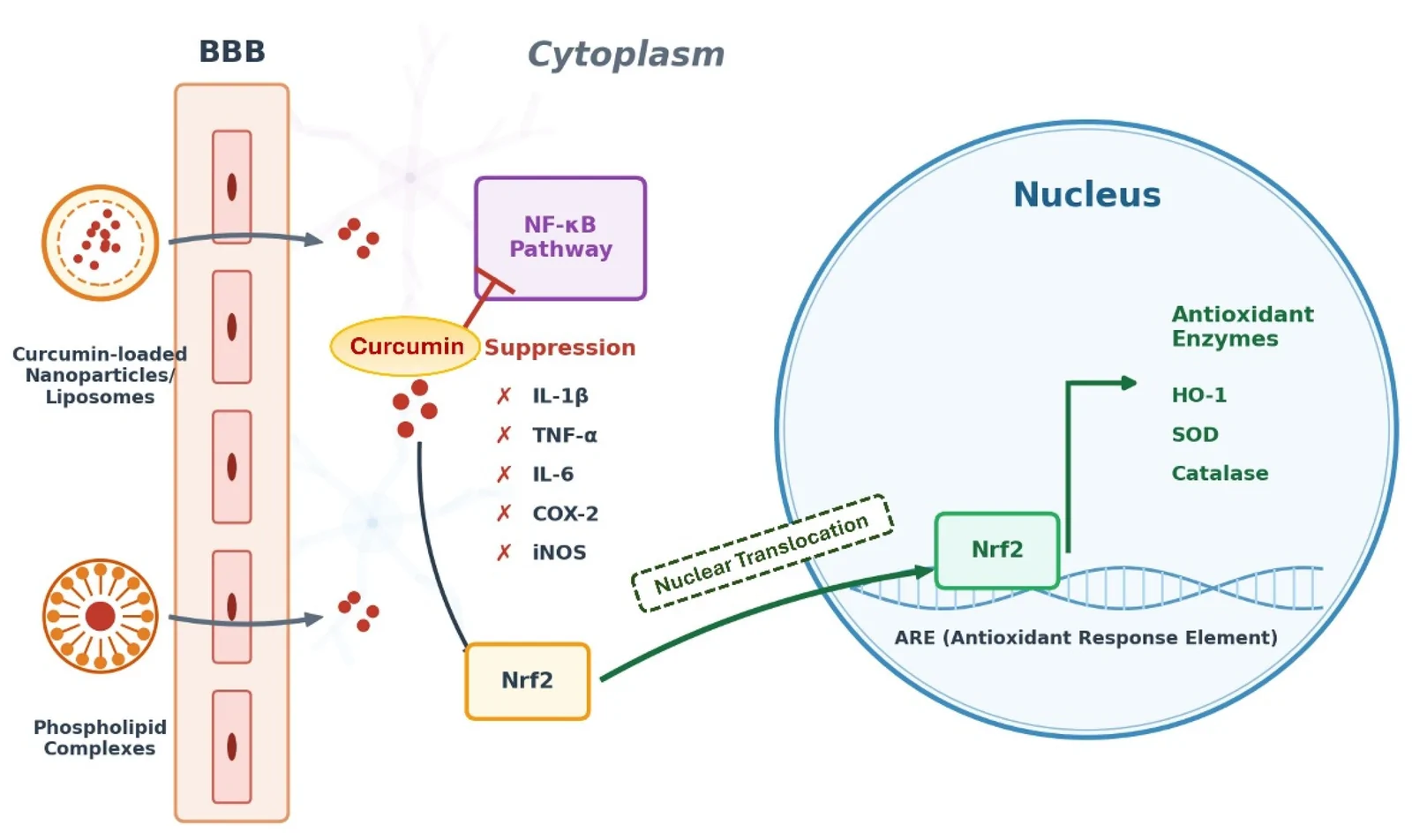
Molecular mechanisms of curcumin-mediated neuroprotection and drug delivery strategies. Schematic overview of curcumin’s multi-target action within the central nervous system. When encapsulated in innovative delivery systems such as nanoparticles, liposomes, and phospholipid complexes, curcumin effectively bypasses the BBB. Once intracellular, it exerts a dual protective effect: Inhibition of the NF-κB Pathway: Curcumin suppresses pro-inflammatory signaling, leading to the downregulation of cytokines (IL-1β, TNF-α, IL-6) and inflammatory enzymes like COX-2 and iNOS. Activation of the Nrf2/ARE Pathway: Curcumin promotes the nuclear translocation of Nrf2, which binds to ARE to induce the expression of cytoprotective enzymes, including HO-1, SOD, and Catalase. Abbreviations: blood–brain barrier (BBB); interleukin-1β (IL-1β); tumor-necrosis factor-alpha (TNF-α); interleukin-6 (IL-6); cyclooxygenase-2 (COX-2); inducible nitric oxide synthase (iNOS); Nuclear Factor Erythroid 2-Related Factor 2 (NRF2); HO-1, heme oxygenase-1 (HO-1); Superoxide Dismutase (SOD); Antioxidant Response Elements (ARE).

**Figure 3:**
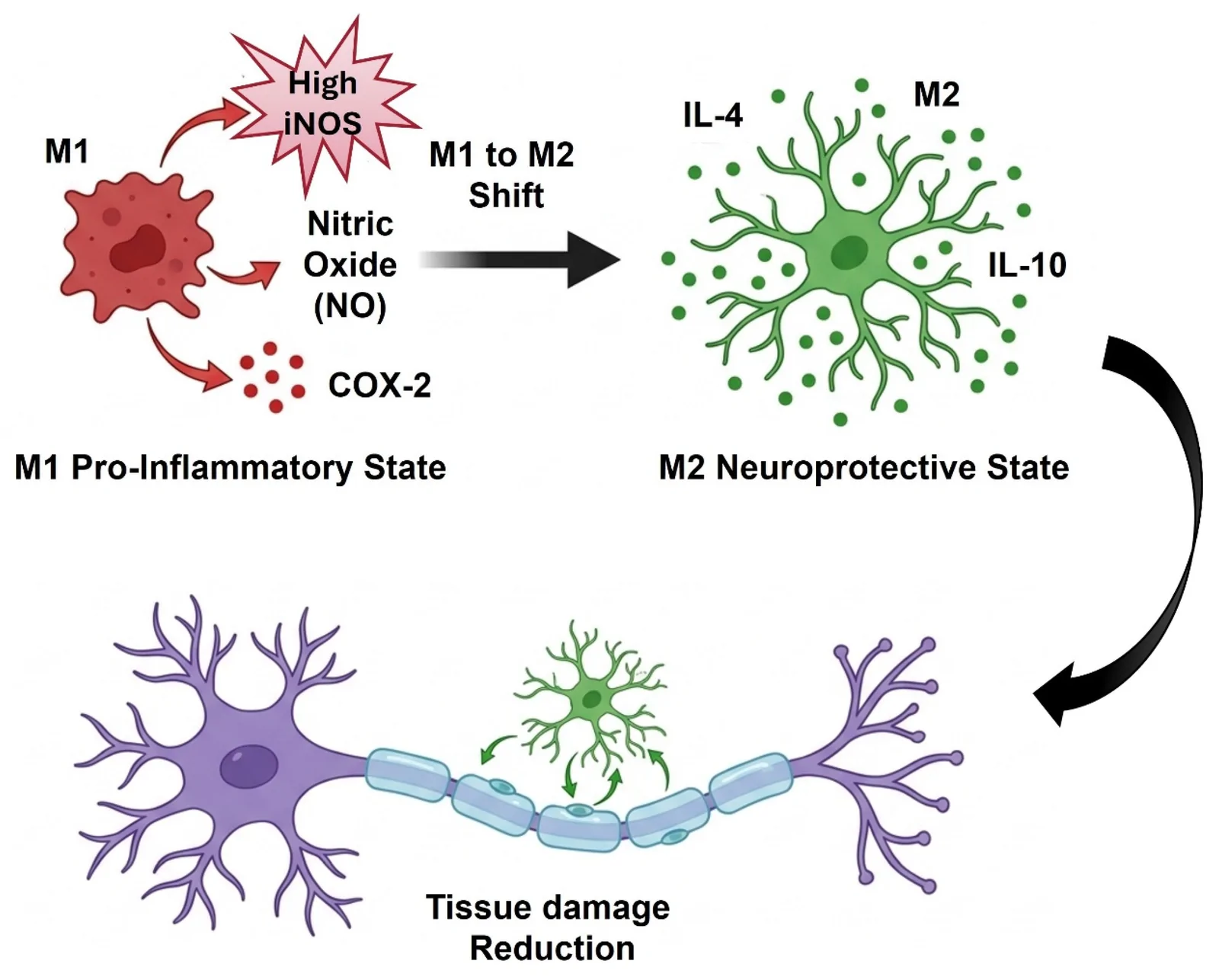
**Curcumin-mediated microglial polarization and the M1 to M2 phenotypic shift.** This illustration details the neuroprotective mechanism of curcumin through the modulation of microglial activation states. Curcumin facilitates the transition from the M1 pro-inflammatory state—characterized by cellular damage and the release of harmful markers such as iNOS, NO, and COX-2—to the M2 neuroprotective state. The M2 phenotype focuses on tissue repair and anti-inflammatory action, promoting the release of beneficial cytokines like IL-4 and IL-10. The overall clinical outcome of this shift is the reversal of chronic cellular stress, the promotion of a state of repair, and the active support of neuronal health while reducing tissue damage. Abbreviations: cyclooxygenase-2 (COX-2); interleukin-4 (IL-4); interleukin-10 (IL-10).

**Figure 4:**
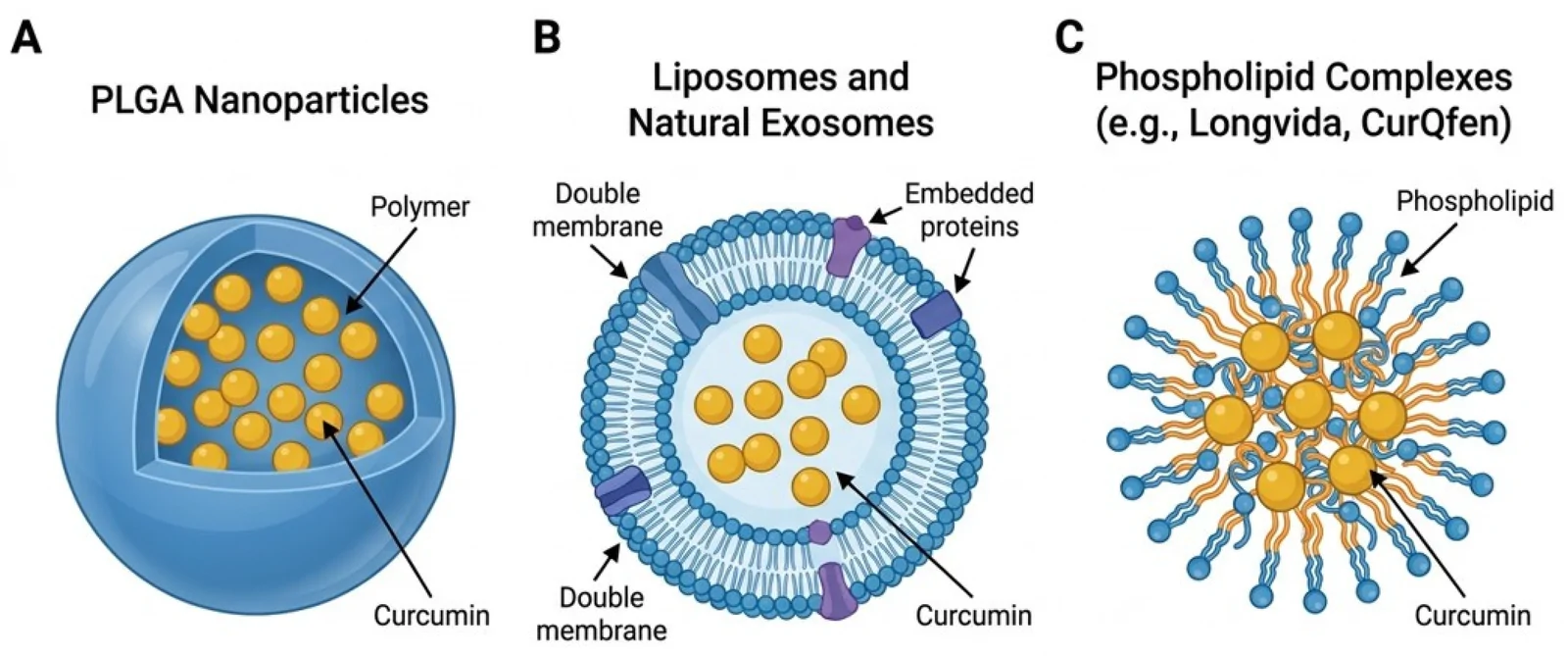
Innovative drug delivery systems for enhanced curcumin bioavailability and brain targeting. This figure illustrates the primary nanotechnology-based strategies employed to overcome curcumin’s inherent limitations, such as poor aqueous solubility and rapid hepatic metabolism. (**A**) PLGA Nanoparticles: Utilization of synthetic biodegradable polymers (PLGA) as controlled-release nanocarriers to improve delivery efficiency and stability (**B**) Liposomes and Natural Exosomes: Spherical lipid bilayers and naturally occurring vesicles (exosomes) designed to mimic cell membranes, thereby facilitating cellular uptake and enhancing the molecular stability of curcumin. (**C**) Phospholipid Complexes (e.g., Longvida, CurQfen): Specialized formulations where curcumin is complexed with phospholipids to drastically increase its ability to cross the blood–brain barrier and achieve effective concentrations within the central nervous system The integration of these delivery vehicles is essential for protecting the molecule from degradation and ensuring effective neuroprotective outcomes in in vivo models Abbreviations: poly lactic-co-glycolic acid (PLGA).

**Table 1:** **In vitro evidence of the role of curcumin in counteracting oxidative stress and neuroinflammation.**

Author/Year	Experimental Context	Main Findings	Ref.
Kang et al., 2004	LPS-stimulated BV-2 microglial cells; COX-2 expression	Curcumin reduced COX-2 expression through inhibition of NF-κB and AP-1 signaling pathways.	[93]
Youn et al., 2006	LPS-induced TLR4 signaling; biochemical and cell-based models	Curcumin-mediated inhibition of TLR4 dimerization and downstream MyD88- and TRIF-dependent signaling.	[90]
Parada et al., 2015	Glial cells exposed to oxidative and inflammatory stress	Promotion of an HO-1-dependent anti-inflammatory phenotype with reduced iNOS expression.	[100]
Porro et al., 2019	BV-2 microglial cells; JAK2/STAT3/SOCS signaling	Increased IL-4 and IL-10 expression, together with modulation of JAK2/STAT3 signaling.	[99]
Bi et al., 2021	U87 glioma cells; HSP60/TLR4/MyD88/NF-κB signaling	Curcumin inhibited the HSP60/TLR4/MyD88/NF-κB pathway, with reduced proliferation and increased apoptosis.	[92]
Jin et al., 2018	Pam3CSK4-stimulated BV-2 microglial cells; inflammatory response	Reduced production of pro-inflammatory mediators and activation of the Nrf2/HO-1 pathway.	[95]
Park et al., 2017	Mn^2+^-exposed BV-2 microglial cells; cytotoxicity and apoptosis	Reduced apoptosis, with decreased Bax expression and caspase-3/-9 activation and increased Bcl-2 levels.	[101]
Zhang et al., 2019	LPS-stimulated BV-2 microglial cells; microglial polarization	Promotion of M2 polarization accompanied by reduced IL-1β and IL-6 production.	[98]
Shi et al., 2021	BV-2 microglial cells; NLRP3 inflammasome activation	Attenuation of microglial activation and IL-1β production through inhibition of NLRP3 inflammasome activation.	[96]
Yin et al., 2018	NLRP3 inflammasome and caspase-1 signaling in an inflammatory cellular model	Inhibition of NLRP3 activation and IL-1β secretion through suppression of caspase-1 activity.	[97]
Yu et al., 2018	LTA-stimulated BV-2 microglial cells; MAPK/NF-κB signaling	Inhibition of MAPK/NF-κB signaling and reduction in pro-inflammatory mediator production.	[94]

**Abbreviations:** Activator protein 1 (AP-1); B-cell lymphoma 2 (Bcl-2); Bcl-2-associated X protein (Bax); cyclooxygenase-2 (COX-2); heat shock protein 60 (HSP60); heme oxygenase-1 (HO-1); interleukin-1β (IL-1β); interleukin-4 (IL-4); interleukin-6 (IL-6); interleukin-10 (IL-10); inducible nitric oxide synthase (iNOS); Janus kinase 2 (JAK2); lipopolysaccharide (LPS); lipoteichoic acid (LTA); mitogen-activated protein kinase (MAPK); myeloid differentiation primary response 88 (MyD88); nuclear factor kappa B (NF-κB); NOD-, LRR- and pyrin domain-containing protein 3 (NLRP3); nuclear factor erythroid 2-related factor 2 (Nrf2); signal transducer and activator of transcription 3 (STAT3); suppressor of cytokine signaling (SOCS); Toll-like receptor 4 (TLR4); TIR-domain-containing adapter-inducing interferon-β (TRIF).

**Table 2:** **In vivo evidence of the neuroprotective and anti-inflammatory effects of curcumin and *Curcuma longa* bioactive compounds.**

Author/Year	Experimental Context	Main Findings	Ref.
He et al., 2010	LPS-exposed neonatal Sprague-Dawley rats; white matter injury; curcumin treatment	Reduced white matter damage and microglial oxidative activation in neonatal rats.	[102]
Zhuang et al., 2011	C57BL/6J mice with experimental autoimmune encephalomyelitis (EAE); exosome-encapsulated curcumin	Attenuation of neuroinflammation and induction of microglial apoptosis.	[103]
Zhang et al., 2017	Rats with subarachnoid hemorrhage (SAH); PLGA-based curcumin nanoparticles; BBB dysfunction	Preservation of BBB integrity with reduced oxidative stress and inflammation.	[104]
Tripanichkul et al., 2013	6-OHDA-induced mouse model of Parkinsonian neurodegeneration	Protection of nigrostriatal dopaminergic neurons and reduction in astrocytic and microglial activation.	[105]
Liu et al., 2017	Experimental ischemic stroke model; microglial polarization	Promotion of M2 polarization, accompanied by reduced inflammation and neuronal damage.	[106]
Zhu et al., 2014	Experimental traumatic brain injury model; TLR4/MyD88/NF-κB signaling	Reduced microglial activation and pro-inflammatory cytokine production through inhibition of the TLR4/MyD88/NF-κB pathway.	[91]
Jiang et al., 2023	Rat spinal cord injury model; transplantation of curcumin-activated olfactory ensheathing cells (aOECs)	Improved functional recovery with promotion of M2-like microglial polarization via APOE/TREM2/NF-κB signaling.	[107]
Wu et al., 2024	Rat spinal cord ischemia–reperfusion injury model; curcumin treatment	Reduced oxidative stress and neuroinflammation through modulation of the Nrf2/NF-κB signaling axis.	[108]
Guo et al., 2024	Spinal cord injury model; transplantation of curcumin-treated olfactory ensheathing cells (OECs)	Enhanced functional recovery associated with IL-4-mediated M2 microglial polarization.	[109]
Ying Liu et al. 2024	C57BL/6 mice with cerebral ischemia–reperfusion induced by middle cerebral artery occlusion (MCAO); curcumol treatment	Reduced infarct volume, neuronal damage, and neuroinflammation, with modulation of microglial polarization and Nrf2/HO-1 and NF-κB signaling.	[110]

**Abbreviations:** apolipoprotein E (APOE); blood–brain barrier (BBB); 6-hydroxydopamine (6-OHDA); experimental autoimmune encephalomyelitis (EAE); interleukin-4 (IL-4); lipopolysaccharide (LPS); middle cerebral artery occlusion (MCAO); myeloid differentiation primary response 88 (MyD88); nuclear factor kappa B (NF-κB); nuclear factor erythroid 2-related factor 2 (Nrf2); olfactory ensheathing cells (OECs); curcumin-activated olfactory ensheathing cells (aOECs); poly(lactic-co-glycolic acid) (PLGA); subarachnoid hemorrhage (SAH); Toll-like receptor 4 (TLR4); triggering receptor expressed on myeloid cells 2 (TREM2).

**Table 3:** **Clinical studies on the neuroprotective potential of curcumin and curcumin-based formulations.**

Authors/Year	Experimental Context	Main Findings	Ref.
Small et al., 2018	Non-demented adults aged 51–84 years; Theracurmin 90 mg twice daily; 18-month randomized, double-blind, placebo-controlled trial	Improved verbal and visual memory and attention, accompanied by reduced amyloid-β and tau tracer uptake in specific brain regions.	[66]
Cox et al., 2015	Healthy adults aged 50–80 years; Longvida 400 mg/day; acute and 28-day supplementation	Improved working memory and sustained attention, with reduced fatigue, anxiety, and stress reactivity following chronic supplementation.	[65]
Hishikawa et al., 2012	Three patients with Alzheimer’s disease; oral turmeric treatment; case report.	Improved behavioral and neuropsychiatric symptoms, with additional positive trends in general cognitive function.	[111]
Baum et al., 2008	Patients with Alzheimer’s disease; standard curcumin 1 or 4 g/day combined with standardized Ginkgo biloba extract; 6-month randomized, double-blind, placebo-controlled pilot trial	Well tolerated, but no significant improvement in MMSE scores; preliminary changes in amyloid-β aggregation and systemic oxidative biomarkers were observed.	[44]
Ringman et al., 2012	Patients with mild-to-moderate Alzheimer’s disease; Curcumin C3 Complex^®^ up to 4 g/day; 24-week randomized, double-blind, placebo-controlled trial	Good safety and tolerability, but no significant clinical benefit or changes in plasma or cerebrospinal fluid Alzheimer’s disease biomarkers.	[112]
Das et al., 2023	Individuals with mild cognitive impairment due to Alzheimer’s disease; CurQfen 400 mg twice daily; randomized controlled trial	Improved executive and neurocognitive performance, together with favorable changes in biomarkers associated with neurodegeneration.	[67]
Ghodsi et al., 2022	Patients with idiopathic Parkinson’s disease; nanomicellar curcumin 80 mg/day; 9-month triple-blind, randomized, placebo-controlled trial	No significant improvement in motor or non-motor outcomes or quality of life compared with placebo.	[113]
Donadio et al., 2022	Patients with idiopathic Parkinson’s disease; oral curcumin supplementation; clinical and skin biopsy study	Improved non-motor symptoms and reduced phosphorylated α-synuclein accumulation in cutaneous autonomic nerves.	[45]
Boshagh et al., 2023	Patients with ischemic stroke undergoing rehabilitation; curcumin-piperine supplementation; 12-week randomized controlled trial	Reduced systemic inflammation and oxidative stress and improved several cardiometabolic parameters.	[43]

**Abbreviations**: Mini-Mental State Examination (MMSE).

## Data Availability

No new data were created or analyzed in this study. Data sharing is not applicable to this article.

## Abbreviations

AD	Alzheimer’s disease
aOECs	olfactory ensheathing cells
BBB	blood–brain barrier
BDMC	bisdemethoxycurcumin
BPSD	behavioral and psychological symptoms of dementia
CIMT	carotid intima-media thickness
CO_2_	carbon dioxide
COX-2	cyclooxygenase-2
CPMAS NMR	cross-polarization magic-angle spinning nuclear magnetic resonance
CUS	curcuminoid Synthase
DESs	deep eutectic solvents
DMC	demethoxycurcumin
EAE	Enzyme-assisted extraction
EFSA	European Food Safety Authority
EMA	European Medicines Agency
FDA	Food and Drug Administration
Fe^2+^	iron
GAMs	glioma-associated microglia/macrophages
GBM	glioblastoma multiforme
GIPAW	gauge-including projector augmented-wave
HDAC	histone deacetylase
HO-1	heme oxygenase-1
HPLC	High-performance liquid chromatography
HPCCC	high-performance countercurrent chromatography
HPTLC	high-performance thin-layer chromatography
hs-CRP	high-sensitivity C-reactive protein
IL-10	interleukin-10
IL-1β	interleukin-1β
IL-4	interleukin-4
iNOS	inducible nitric oxide synthase
IRMS	isotope-ratio mass spectrometry
LPS	lipopolysaccharide
LTA	Lipoteichoic acid
MACs	monocarbonyl analogs of curcumin
MAPK	Mitogen-activated protein kinase
Mn^2+^	manganese
NADESs	natural deep eutectic solvents
NF-κB	nuclear factor-kappa B
NMR	nuclear magnetic resonance (NMR) spectroscopy
O_2_^−^	superoxide anion
OH	hydroxyl radical
PAL	Phenylalanine ammonia-Lyase
PD	Parkinson’s disease
PLE	Pressurized Liquid Extraction
qNMR	quantitative nuclear magnetic resonance
RCTs	randomized controlled trials
ROS	reactive oxygen species
SAS	Supercritical AntiSolvent
SCFA	short-chain fatty acid
SLNs	Solid lipid nanoparticles
SRT	Buschke Selective Reminding Test
TAC	Total Antioxidant Capacity
TGF-β	transforming growth factor-beta
THC	tetrahydrocurcumin
TLR4	Toll-like receptor 4
TNF-α	tumor-necrosis factor-alpha
UGT	UDP-glucuronosyltransferase
Zn^2+^	metal ions like zinc
ZO-1	zonula occludens-1

## References

[1] Slaček G., Kotnik P., Osmić A., Postružnik V., Knez Ž., Finšgar M., Knez Marevci M. (2023). The Extraction Process, Separation, and Identification of Curcuminoids from Turmeric *Curcuma longa*. Foods.

[2] Jiang T., Ghosh R., Charcosset C. (2021). Extraction, purification and applications of curcumin from plant materials-A comprehensive review. Trends Food Sci. Technol..

[3] Binello A., Grillo G., Barge A., Allegrini P., Ciceri D., Cravotto G. (2020). A Cross-Flow Ultrasound-Assisted Extraction of Curcuminoids from *Curcuma longa* L.: Process Design to Avoid Degradation. Foods.

[4] Vardhini N.M., Punia J., Jat S., Pawar S.D., Devi N., Radhakrishnanand P., Murty U.S., Saini A., Sethi K.K., Kumar P. (2023). Purification and Characterization of Pure Curcumin, Desmethoxycurcumin, and Bisdesmethoxycurcumin from North-East India Lakadong Turmeric (*Curcuma longa*). J. Chromatogr. A.

[5] Łubek-Nguyen A., Ziemichód W., Olech M. (2022). Application of Enzyme-Assisted Extraction for the Recovery of Natural Bioactive Compounds for Nutraceutical and Pharmaceutical Applications. Appl. Sci..

[6] Tripathy S., Verma D.K., Thakur M., Patel A.R., Srivastav P.P., Singh S., Gupta A.K., Chávez-González M.L., Aguilar C.N., Chakravorty N. (2021). Curcumin Extraction, Isolation, Quantification and Its Application in Functional Foods: A Review with a Focus on Immune Enhancement Activities and COVID-19. Front. Nutr..

[7] Mandal V., Tandey R. (2016). A critical analysis of publication trends from 2005–2015 in microwave assisted extraction of botanicals: How far we have come and the road ahead. TrAC Trends Anal. Chem..

[8] Lianza M., Marincich L., Antognoni F. (2022). The Greening of Anthocyanins: Eco-Friendly Techniques for Their Recovery from Agri-Food By-Products. Antioxidants.

[9] Usman I., Hussain M., Imran A., Afzaal M., Saeed F., Javed M., Afzal A., Ashfaq I., Al Jbawi E., Saewan S.A. (2022). Traditional and innovative approaches for the extraction of bioactive compounds. Int. J. Food Prop..

[10] Wassie T., Niu K., Xie C., Wang H., Xin W. (2021). Extraction Techniques, Biological Activities and Health Benefits of Marine Algae Enteromorpha prolifera Polysaccharide. Front. Nutr..

[11] Widmann A.-K., Wahl M.A., Kammerer D.R., Daniels R. (2022). Supercritical Fluid Extraction with CO_2_ of *Curcuma longa* L. in Comparison to Conventional Solvent Extraction. Pharmaceutics.

[12] Gopalan B., Goto M., Kodama A., Hirose T. (2000). Supercritical carbon dioxide extraction of turmeric (*Curcuma longa*). J. Agric. Food Chem..

[13] Kapadia P., Newell A.S., Cunningham J., Roberts M.R., Hardy J.G. (2022). Extraction of High-Value Chemicals from Plants for Technical and Medical Applications. Int. J. Mol. Sci..

[14] Li Y., Qaria M.A., Sivasamy S., Sun J., Zhu D. (2021). Curcumin production and bioavailability: A comprehensive review of curcumin extraction, synthesis, biotransformation and delivery systems. Ind. Crops Prod..

[15] Pyo D., Eojin K. (2014). Rapid and Efficient Extraction of Curcumins from Curry Powder Using Supercritical CO_2_. Bull. Korean Chem. Soc..

[16] Chemat F., Vian M.A., Fabiano-Tixier A.S., Nutrizio M., Režek Jambrak A., Munekata P.E.S., Lorenzo J.M., Barba F.J., Binello A., Cravotto G. (2020). A Review of Sustainable and Intensified Techniques for Extraction of Food and Natural Products. Green Chem..

[17] Wu X., Huang Q., Mao Y., Wang X., Wang Y., Hu Q., Wang H., Wang X. (2019). Sensors for Determination of Uranium: A Review. TrAC Trends Anal. Chem..

[18] Choma A., Nowak K., Komaniecka I., Waśko A., Pleszczyńska M., Siwulski M., Wiater A. (2018). Chemical characterization of alkali-soluble polysaccharides isolated from a Boletus edulis (Bull.) fruiting body and their potential for heavy metal biosorption. Food Chem..

[19] Azmir J., Zaidul I.S.M., Rahman M.M., Sharif K.M., Mohamed A., Sahena F., Jahurul M.H.A., Ghafoor K., Norulaini N.A.N., Omar A.K.M. (2013). Techniques for Extraction of Bioactive Compounds from Plant Materials: A Review. J. Food Eng..

[20] Li H., Zhang C., Pang C., Li X., Gao X. (2020). The Advances in the Special Microwave Effects of the Heterogeneous Catalytic Reactions. Front. Chem..

[21] Singh K., Srichairatanakool S., Chewonarin T., Prommaban A., Samakradhamrongthai R.S., Brennan M.A., Brennan C.S., Utama-ang N. (2022). Impact of Green Extraction on Curcuminoid Content, Antioxidant Activities and Anti-Cancer Efficiency (In Vitro) from Turmeric Rhizomes (*Curcuma longa* L.). Foods.

[22] Fernández-Marín R., Fernandes S.C.M., Andrés M.A., Labidi J. (2021). Microwave-Assisted Extraction of *Curcuma longa* L. Oil: Optimization, Chemical Structure and Composition, Antioxidant Activity and Comparison with Conventional Soxhlet Extraction. Molecules.

[23] Wang C., Yang H., Li J. (2021). Combination of Microwave, Ultrasonic, Enzyme Assisted Method for Curcumin Species Extraction from Turmeric (*Curcuma longa* L.) and Evaluation of their Antioxidant Activity. eFood.

[24] Jovanović J., Jović M., Trifković J., Smiljanić K., Gašić U., Krstić Ristivojević M., Ristivojević P. (2025). Green Extraction of Bioactives from *Curcuma longa* Using Natural Deep Eutectic Solvents: Unlocking Antioxidative, Antimicrobial, Antidiabetic, and Skin Depigmentation Potentials. Plants.

[25] Sai Lakshmi-Manasa P., Alka D., Chilakamarthi U.-C. (2023). Various Extraction Techniques of Curcumin: A Comprehensive Review. ACS Omega.

[26] Valizadeh Kiamahalleh M., Najafpour-Darzi G., Rahimnejad M., Moghadamnia A.A. (2016). High performance curcumin subcritical water extraction from turmeric (*Curcuma longa* L.). J. Chromatogr. B.

[27] Jeliński T., Przybyłek M., Cysewski P. (2019). Natural Deep Eutectic Solvents as Agents for Improving Solubility, Stability and Delivery of Curcumin. Pharm. Res..

[28] Dai Y., van Spronsen J., Witkamp G.-J., Verpoorte R., Choi Y.H. (2013). Ionic liquids and deep eutectic solvents in natural products research: Mixtures of solids as extraction solvents. J. Nat. Prod..

[29] Kimthet C., Chhouk K., Diono W., Kanda H., Goto M. (2017). Extraction of curcumin from *Curcuma longa* L. using ultrasound assisted supercritical carbon dioxide. AIP Conf. Proc..

[30] Xu G., Hao C., Tian S., Gao F., Sun W., Sun R. (2017). A method for the preparation of curcumin by ultrasonic-assisted ammonium sulfate/ethanol aqueous two-phase extraction. J. Chromatogr. B.

[31] Yang Z., Zheng S., Rui Z., Fang Y., Ji H. (2015). Enhanced separation and purification of curcuminoids on polyamide column via noncovalent interactions. Sep. Purif. Technol..

[32] Inoue K., Nomura C., Ito S., Nagatsu A., Hino T., Oka H. (2008). Purification of curcumin, demethoxycurcumin, and bisdemethoxycurcumin by high-speed countercurrent chromatography. J. Agric. Food Chem..

[33] Kita T., Imai S., Sawada H., Kumagai H., Seto H. (2008). The Biosynthetic Pathway of Curcuminoid in Turmeric (*Curcuma longa*) as Revealed by 13C-Labeled Precursors. Biosci. Biotechnol. Biochem..

[34] Chakraborty A., Mahajan S., Jaiswal S.K., Sharma V.K. (2021). Genome Sequencing of Turmeric Provides Evolutionary insights into Its Medicinal Properties. Commun. Biol..

[35] Pabon H.J.J. (1964). A synthesis of curcumin and related compounds. Recl. Trav. Chim..

[36] Kim B.R., Park J.Y., Jeong H.J., Kwon H.J., Park S.J., Lee I.C., Ryu Y.B., Lee W.S. (2018). Design, Synthesis, and Evaluation of Curcumin Analogues as Potential Inhibitors of Bacterial Sialidase. J. Enzyme Inhib. Med. Chem..

[37] Priyadarsini K.I. (2014). The Chemistry of Curcumin: From Extraction to Therapeutic Agent. Molecules.

[38] Chainoglou E., Hadjipavlou-Litina D. (2019). Curcumin analogues and derivatives with anti-proliferative and anti-inflammatory activity: Structural characteristics and molecular targets. Expert Opin. Drug Discov..

[39] Wei T., Zheng Z., Wei X., Liu Y., Li W., Fang B., Yun D., Dong Z., Yi B., Li W. (2022). Rational design, synthesis, and pharmacological characterisation of dicarbonyl curcuminoid analogues with improved stability against lung cancer via ROS and ER stress mediated cell apoptosis and pyroptosis. J. Enzyme Inhib. Med. Chem..

[40] Nagargoje A.A., Akolkar S.V., Siddiqui M.M., Subhedar D.D., Sangshetti J.N., Khedkar V.M., Shingate B.B. (2020). Quinoline Based Monocarbonyl Curcumin Analogs as Potential Antifungal and Antioxidant Agents: Synthesis, Bioevaluation and Molecular Docking Study. Chem. Biodivers..

[41] Mary C.P.V., Vijayakumar S., Shankar R. (2018). Metal chelating ability and antioxidant properties of Curcumin-metal complexes—A DFT approach. J. Mol. Graph. Model..

[42] Shoba G., Joy D., Joseph T., Majeed M., Rajendran R., Srinivas P.S. (1998). Influence of piperine on the pharmacokinetics of curcumin in animals and human volunteers. Planta Med..

[43] Boshagh K., Khorvash F., Sahebkar A., Majeed M., Bahreini N., Askari G., Bagherniya M. (2023). The effects of curcumin-piperine supplementation on inflammatory, oxidative stress and metabolic indices in patients with ischemic stroke in the rehabilitation phase: A randomized controlled trial. Nutr. J..

[44] Baum L., Lam C.W., Cheung S.K., Kwok T., Lui V., Tsoh J., Lam L., Leung V., Hui E., Ng C. (2008). Six-month randomized, placebo-controlled, double-blind, pilot clinical trial of curcumin in patients with Alzheimer disease. J. Clin. Psychopharmacol..

[45] Donadio V., Incensi A., Rizzo G., Fileccia E., Ventruto F., Riva A., Tiso D., Recchia M., Vacchiano V., Infante R. (2022). The Effect of Curcumin on Idiopathic Parkinson Disease: A Clinical and Skin Biopsy Study. J. Neuropathol. Exp. Neurol..

[46] Iweala E.J., Uche M.E., Dike E.D., Etumnu L.R., Dokunmu T.M., Oluwapelumi A.E., Okoro B.C., Dania O.E., Adebayo A.H., Ugbogu E.A. (2023). *Curcuma longa* (Turmeric): Ethnomedicinal Uses, Phytochemistry, Pharmacological Activities and Toxicity Profiles—A Review. Pharmacol. Res. Mod. Chin. Med..

[47] Katsuyama Y., Kita T., Funa N., Horinouchi S. (2009). Curcuminoid biosynthesis by two type III polyketide synthases in the herb *Curcuma longa*. J. Biol. Chem..

[48] Liu C., Li S. (2022). Corrigendum: Engineered biosynthesis of plant polyketides by type III polyketide synthases in microorganisms. Front. Bioeng. Biotechnol..

[49] Abe I. (2020). Biosynthesis of medicinally important plant metabolites by unusual type III polyketide synthases. J. Nat. Med..

[50] Katsuyama Y., Kita T., Horinouchi S. (2009). Identification and characterization of multiple curcumin synthases from the herb *Curcuma longa*. FEBS Lett..

[51] Bandyopadhyay D. (2014). Farmer to pharmacist: Curcumin as an anti-invasive and antimetastatic agent for the treatment of cancer. Front. Chem..

[52] Qiu B., Xu X., Yi P., Hao Y. (2020). Curcumin reinforces MSC-derived exosomes in attenuating osteoarthritis via modulating the miR-124/NF-kB and miR-143/ROCK1/TLR9 signalling pathways. J. Cell Mol. Med..

[53] Ren B.C., Zhang Y.F., Liu S.S., Cheng X.J., Yang X., Cui X.G., Zhao X.R., Zhao H., Hao M.F., Li M.D. (2020). Curcumin alleviates oxidative stress and inhibits apoptosis in diabetic cardiomyopathy via Sirt1-Foxo1 and PI3K-Akt signalling pathways. J. Cell Mol. Med..

[54] Shamsnia H.S., Roustaei M., Ahmadvand D., Butler A.E., Amirlou D., Soltani S., Momtaz S., Jamialahmadi T., Abdolghaffari A.H., Sahebkar A. (2023). Impact of curcumin on p38 MAPK: Therapeutic implications. Inflammopharmacology.

[55] Fabianowska-Majewska K., Kaufman-Szymczyk A., Szymanska-Kolba A., Jakubik J., Majewski G., Lubecka K. (2021). Curcumin from Turmeric Rhizome: A Potential Modulator of DNA Methylation Machinery in Breast Cancer Inhibition. Nutrients.

[56] Yang L., Chen X., Bi Z., Liao J., Zhao W., Huang W. (2021). Curcumin attenuates renal ischemia reperfusion injury via JNK pathway with the involvement of p300/CBP-mediated histone acetylation. Korean J. Physiol. Pharmacol..

[57] Ming T., Tao Q., Tang S., Zhao H., Yang H., Liu M., Ren S., Xu H. (2022). Curcumin: An epigenetic regulator and its application in cancer. Biomed. Pharmacother..

[58] Bučević Popović V., Karahmet Farhat E., Banjari I., Jeličić Kadić A., Puljak L. (2024). Bioavailability of Oral Curcumin in Systematic Reviews: A Methodological Study. Pharmaceuticals.

[59] Dei Cas M., Ghidoni R. (2019). Dietary Curcumin: Correlation between Bioavailability and Health Potential. Nutrients.

[60] Drion C.M., Borm L.E., Kooijman L., Aronica E., Wadman W.J., Hartog A.F., van Vliet E.A., Gorter J.A. (2016). Effects of rapamycin and curcumin treatment on the development of epilepsy after electrically induced status epilepticus in rats. Epilepsia.

[61] Chen Y., Lu Y., Lee R.J., Xiang G. (2020). Nano Encapsulated Curcumin: And Its Potential for Biomedical Applications. Int. J. Nanomed..

[62] Quispe C., Cruz-Martins N., Manca M.L., Manconi M., Sytar O., Hudz N., Shanaida M., Kumar M., Taheri Y., Martorell M. (2021). Nano-Derived Therapeutic Formulations with Curcumin in Inflammation-Related Diseases. Oxid. Med. Cell Longev..

[63] Hassanizadeh S., Shojaei M., Bagherniya M., Orekhov A.N., Sahebkar A. (2023). Effect of nano-curcumin on various diseases: A comprehensive review of clinical trials. Biofactors.

[64] Mohammadzadeh R., Fathi M., Pourseif M.M., Omidi Y., Farhang S., Barzegar Jalali M., Valizadeh H., Nakhlband A., Adibkia K. (2024). Curcumin and nano-curcumin applications in psychiatric disorders. Phytother. Res..

[65] Cox K.H., Pipingas A., Scholey A.B. (2015). Investigation of the effects of solid lipid curcumin on cognition and mood in a healthy older population. J. Psychopharmacol..

[66] Small G.W., Siddarth P., Li Z., Miller K.J., Ercoli L., Emerson N.D., Martinez J., Wong K.P., Liu J., Merrill D.A. (2018). Memory and Brain Amyloid and Tau Effects of a Bioavailable Form of Curcumin in Non-Demented Adults: A Double-Blind, Placebo-Controlled 18-Month Trial. Am. J. Geriatr. Psychiatry.

[67] Das S.S., Gopal P.M., Thomas J.V., Mohan M.C., Thomas S.C., Maliakel B.P., Krishnakumar I.M., Pulikkaparambil Sasidharan B.C. (2023). Influence of CurQfen^®^-curcumin on cognitive impairment: A randomized, double-blinded, placebo-controlled, 3-arm, 3-sequence comparative study. Front. Dement..

[68] Concetta Scuto M., Mancuso C., Tomasello B., Laura Ontario M., Cavallaro A., Frasca F., Maiolino L., Trovato Salinaro A., Calabrese E.J., Calabrese V. (2019). Curcumin, Hormesis and the Nervous System. Nutrients.

[69] Wei L., Ren H., Zhao M., Xu T., Yang J., Deng X., Sun J., Zhou S., Zhang J., Gao X. (2026). Curcumin reduces neuroinflammation and oxidative stress in a stroke model by epigenetically regulating ADRB2 methylation through JAK2/STAT3 and Nrf2/HO-1 pathways. J. Neuroinflammation.

[70] Omolekan T.O., Chamcheu J.C., Buerger C., Huang S. (2024). PI3K/AKT/mTOR Signaling Network in Human Health and Diseases. Cells.

[71] Wong M. (2013). Mammalian target of rapamycin (mTOR) pathways in neurological diseases. Biomed. J..

[72] Liu M., Wang J., Song Z., Pei Y. (2025). Regulation mechanism of curcumin mediated inflammatory pathway and its clinical application: A review. Front. Pharmacol..

[73] Moldoveanu C.-A., Tomoaia-Cotisel M., Sevastre-Berghian A., Tomoaia G., Mocanu A., Pal-Racz C., Toma V.-A., Roman I., Ujica M.-A., Pop L.-C. (2025). A Review on Current Aspects of Curcumin-Based Effects in Relation to Neurodegenerative, Neuroinflammatory and Cerebrovascular Diseases. Molecules.

[74] Fornari Laurindo L., Aparecido Dias J., Cressoni Araújo A., Torres Pomini K., Machado Galhardi C., Rucco Penteado Detregiachi C., Santos de Argollo Haber L., Donizeti Roque D., Dib Bechara M., Vialogo Marques de Castro M. (2024). Immunological dimensions of neuroinflammation and microglial activation: Exploring innovative immunomodulatory approaches to mitigate neuroinflammatory progression. Front. Immunol..

[75] Becher B., Derfuss T., Liblau R. (2024). Targeting cytokine networks in neuroinflammatory diseases. Nat. Rev. Drug Discov..

[76] Priyadarsini K.I., Maity D.K., Naik G.H., Kumar M.S., Unnikrishnan M.K., Satav J.G., Mohan H. (2003). Role of phenolic O-H and methylene hydrogen on the free radical reactions and antioxidant activity of curcumin. Free Radic. Biol. Med..

[77] Feng J.Y., Liu Z.Q. (2009). Phenolic and enolic hydroxyl groups in curcumin: Which plays the major role in scavenging radicals?. J. Agric. Food Chem..

[78] Vasconcelos A.R., Dos Santos N.B., Scavone C., Munhoz C.D. (2019). Nrf2/ARE Pathway Modulation by Dietary Energy Regulation in Neurological Disorders. Front. Pharmacol..

[79] Mayer C., Riera-Ponsati L., Kauppinen S., Klitgaard H., Erler J.T., Hansen S.N. (2024). Targeting the NRF2 pathway for disease modification in neurodegenerative diseases: Mechanisms and therapeutic implications. Front. Pharmacol..

[80] Di Meo F., Margarucci S., Galderisi U., Crispi S., Peluso G. (2019). Curcumin, Gut Microbiota, and Neuroprotection. Nutrients.

[81] Enayati A., Soghi A., Butler A.E., Rizzo M., Sahebkar A. (2023). The Effect of Curcumin on the Gut-Brain Axis: Therapeutic Implications. J. Neurogastroenterol. Motil..

[82] Cerullo M., Armeli F., Mengoni B., Menin M., Crudeli M.L., Businaro R. (2025). Curcumin Modulation of the Gut–Brain Axis for Neuroinflammation and Metabolic Disorders Prevention and Treatment. Nutrients.

[83] Ghosh S.S., He H., Wang J., Gehr T.W., Ghosh S. (2018). Curcumin-mediated regulation of intestinal barrier function: The mechanism underlying its beneficial effects. Tissue Barriers.

[84] Zhu J., He L. (2024). The Modulatory Effects of Curcumin on the Gut Microbiota: A Potential Strategy for Disease Treatment and Health Promotion. Microorganisms.

[85] Sun J., Xie Q., Sun M., Zhang W., Wang H., Liu N., Wang M. (2024). Curcumin protects mice with myasthenia gravis by regulating the gut microbiota, short-chain fatty acids, and the Th17/Treg balance. Heliyon.

[86] Sun Z.Z., Li X.Y., Wang S., Shen L., Ji H.F. (2020). Bidirectional interactions between curcumin and gut microbiota in transgenic mice with Alzheimer’s disease. Appl. Microbiol. Biotechnol..

[87] Mansouri R., Mousavi S.N., Shokri R., Afshar D., Shapouri R. (2025). Curcumin supplementation increases butyrogenic bacteria in the gut of obese individuals following a weight-loss diet: A randomized controlled trial. J. Funct. Foods.

[88] Lively S., Schlichter L.C. (2018). Microglia Responses to Pro-inflammatory Stimuli (LPS, IFNγ+TNFα) and Reprogramming by Resolving Cytokines (IL-4, IL-10). Front. Cell. Neurosci..

[89] Devanney N.A., Stewart A.N., Gensel J.C. (2020). Microglia and macrophage metabolism in CNS injury and disease: The role of immunometabolism in neurodegeneration and neurotrauma. Exp. Neurol..

[90] Youn H.S., Saitoh S.I., Miyake K., Hwang D.H. (2006). Inhibition of homodimerization of Toll-like receptor 4 by curcumin. Biochem. Pharmacol..

[91] Zhu H.T., Bian C., Yuan J.C., Chu W.H., Xiang X., Chen F., Wang C.S., Feng H., Lin J.K. (2014). Curcumin attenuates acute inflammatory injury by inhibiting the TLR4/MyD88/NF-κB signaling pathway in experimental traumatic brain injury. J. Neuroinflamm..

[92] Bi F., Wang J., Zheng X., Xiao J., Zhi C., Gu J., Zhang Y., Li J., Miao Z., Wang Y. (2021). HSP60 participates in the anti-glioma effects of curcumin. Exp. Ther. Med..

[93] Kang G., Kong P.J., Yuh Y.J., Lim S.Y., Yim S.V., Chun W., Kim S.S. (2004). Curcumin suppresses lipopolysaccharide-induced cyclooxygenase-2 expression by inhibiting activator protein 1 and nuclear factor kappab bindings in BV2 microglial cells. J. Pharmacol. Sci..

[94] Yu Y., Shen Q., Lai Y., Park S.Y., Ou X., Lin D., Jin M., Zhang W. (2018). Anti-inflammatory Effects of Curcumin in Microglial Cells. Front. Pharmacol..

[95] Jin M., Park S.Y., Shen Q., Lai Y., Ou X., Mao Z., Lin D., Yu Y., Zhang W. (2018). Anti-neuroinflammatory effect of curcumin on Pam3CSK4-stimulated microglial cells. Int. J. Mol. Med..

[96] Shi Q., Zheng Y., Wang L., Xue Y., Yang Y. (2021). Curcumin suppresses neuroinflammation to protect neurons by preventing NLRP3 inflammasome activation. Eur. J. Inflamm..

[97] Yin H., Guo Q., Li X., Tang T., Li C., Wang H., Sun Y., Feng Q., Ma C., Gao C. (2018). Curcumin Suppresses IL-1β Secretion and Prevents Inflammation through Inhibition of the NLRP3 Inflammasome. J. Immunol..

[98] Zhang J., Zheng Y., Luo Y., Du Y., Zhang X., Fu J. (2019). Curcumin inhibits LPS-induced neuroinflammation by promoting microglial M2 polarization via TREM2/TLR4/NF-κB pathways in BV2 cells. Mol. Immunol..

[99] Porro C., Cianciulli A., Trotta T., Lofrumento D.D., Panaro M.A. (2019). Curcumin Regulates Anti-Inflammatory Responses by JAK/STAT/SOCS Signaling Pathway in BV-2 Microglial Cells. Biology.

[100] Parada E., Buendia I., Navarro E., Avendaño C., Egea J., López M.G. (2015). Microglial HO-1 induction by curcumin provides antioxidant, antineuroinflammatory, and glioprotective effects. Mol. Nutr. Food Res..

[101] Park E., Chun H.S. (2017). Protective Effects of Curcumin on Manganese-Induced BV-2 Microglial Cell Death. Biol. Pharm. Bull..

[102] He L.F., Chen H.J., Qian L.H., Chen G.Y., Buzby J.S. (2010). Curcumin protects pre-oligodendrocytes from activated microglia in vitro and in vivo. Brain Res..

[103] Zhuang X., Xiang X., Grizzle W., Sun D., Zhang S., Axtell R.C., Ju S., Mu J., Zhang L., Steinman L. (2011). Treatment of brain inflammatory diseases by delivering exosome encapsulated anti-inflammatory drugs from the nasal region to the brain. Mol. Ther..

[104] Zhang Z.Y., Jiang M., Fang J., Yang M.F., Zhang S., Yin Y.X., Li D.W., Mao L.L., Fu X.Y., Hou Y.J. (2017). Enhanced Therapeutic Potential of Nano-Curcumin Against Subarachnoid Hemorrhage-Induced Blood-Brain Barrier Disruption Through Inhibition of Inflammatory Response and Oxidative Stress. Mol. Neurobiol..

[105] Tripanichkul W., Jaroensuppaperch E.O. (2013). Ameliorating effects of curcumin on 6-OHDA-induced dopaminergic denervation, glial response, and SOD1 reduction in the striatum of hemiparkinsonian mice. Eur. Rev. Med. Pharmacol. Sci..

[106] Liu Z., Ran Y., Huang S., Wen S., Zhang W., Liu X., Ji Z., Geng X., Ji X., Du H. (2017). Curcumin Protects against Ischemic Stroke by Titrating Microglia/Macrophage Polarization. Front. Aging Neurosci..

[107] Jiang C., Chen Z., Wang X., Zhang Y., Guo X., Fan H., Huang D., He Y., Tang X., Ai Y. (2023). Curcumin-activated Olfactory Ensheathing Cells Improve Functional Recovery After Spinal Cord Injury by Modulating Microglia Polarization Through APOE/TREM2/NF-κB Signaling Pathway. J. Neuroimmune Pharmacol..

[108] Wu F., Lin Y., Xiao L., Chen Q., Lin F., Li R. (2024). Administration with curcumin alleviates spinal cord ischemia-reperfusion injury by regulating anti-oxidative stress and microglia activation-mediated neuroinflammation via Nrf2/NF-κB axis. In Vitr. Cell. Dev. Biol. Anim..

[109] Guo J., Tang X., Deng P., Hui H., Chen B., An J., Zhang G., Shi K., Wang J., He Y. (2024). Interleukin-4 from curcumin-activated OECs emerges as a central modulator for increasing M2 polarization of microglia/macrophage in OEC anti-inflammatory activity for functional repair of spinal cord injury. Cell Commun. Signal.

[110] Liu Y., Wang W., Di B., Miao J. (2024). Curcumol ameliorates neuroinflammation after cerebral ischemia-reperfusion injury via affecting microglial polarization and Treg/Th17 balance through Nrf2/HO-1 and NF-κB signaling. Cell Death Discov..

[111] Hishikawa N., Takahashi Y., Amakusa Y., Tanno Y., Tuji Y., Niwa H., Murakami N., Krishna U.K. (2012). Effects of turmeric on Alzheimer’s disease with behavioral and psychological symptoms of dementia. AYU.

[112] Ringman J.M., Frautschy S.A., Teng E., Begum A.N., Bardens J., Beigi M., Gylys K.H., Badmaev V., Heath D.D., Apostolova L.G. (2012). Oral curcumin for Alzheimer’s disease: Tolerability and efficacy in a 24-week randomized, double blind, placebo-controlled study. Alzheimers Res. Ther..

[113] Ghodsi H., Rahimi H.R., Aghili S.M., Saberi A., Shoeibi A. (2022). Evaluation of curcumin as add-on therapy in patients with Parkinson’s disease: A pilot randomized, triple-blind, placebo-controlled trial. Clin. Neurol. Neurosurg..

[114] Noorafshan A., Ashkani-Esfahani S. (2013). A Review of Therapeutic Effects of Curcumin. Curr. Pharm. Des..

[115] Li N., Liu T.H., Yu J.Z., Li C.X., Liu Y., Wu Y.Y., Yang Z.S., Yuan J.L. (2019). Curcumin and Curcumol Inhibit NF-κB and TGF-β1/Smads Signaling Pathways in CSE-Treated RAW264.7 Cells. Evid. Based Complement Altern. Med..

[116] Abdel-Daim M.M., Abdou R.H. (2015). Protective Effects of Diallyl Sulfide and Curcumin Separately against Thallium-Induced Toxicity in Rats. Cell J..

[117] Malik P., Hoidal J.R., Mukherjee T.K. (2021). Recent Advances in Curcumin Treated Non-Small Cell Lung Cancers: An Impetus of Pleiotropic Traits and Nanocarrier Aided Delivery. Curr. Med. Chem..

[118] Kunnumakkara A.B., Harsha C., Banik K., Vikkurthi R., Sailo B.L., Bordoloi D., Gupta S.C., Aggarwal B.B. (2019). Is Curcumin Bioavailability a Problem in Humans: Lessons from Clinical Trials. Expert Opin. Drug Metab. Toxicol..

[119] European Food Safety Authority (2014). Refined Exposure Assessment for Curcumin (E 100). EFSA J..

[120] Dwyer J.T., Coates P.M., Smith M.J. (2018). Dietary Supplements: Regulatory Challenges and Research Resources. Nutrients.

[121] Girme A., Saste G., Balasubramaniam A.K., Pawar S., Ghule C., Hingorani L. (2020). Assessment of *Curcuma longa* Extract for Adulteration with Synthetic Curcumin by Analytical Investigations. J. Pharm. Biomed. Anal..

[122] Takikawa H., Murata Y., Horiike N., Fukui H., Onji M. (2009). Drug-Induced Liver Injury in Japan: An Analysis of 1676 Cases between 1997 and 2006. Hepatol. Res..

[123] Lukefahr A.L., McEvoy S., Alfafara C., Funk J.L. (2018). Drug-Induced Autoimmune Hepatitis Associated with Turmeric Dietary Supplement Use. BMJ Case Rep..

[124] Luber R.P., Rentsch C., Lontos S., Pope J.D., Aung A.K., Schneider H.G., Kemp W., Roberts S.K., Majeed A. (2019). Turmeric Induced Liver Injury: A Report of Two Cases. Case Rep. Hepatol..

[125] Committee on Herbal Medicinal Products (HMPC) Assessment report on *Curcuma longa* L., rhizoma. https://www.ema.europa.eu/en/documents/herbal-report/final-assessment-report-curcuma-longa-l-rhizoma-revision-1_en.pdf.

[126] Bucchini L. (2019). The 2019 Curcumin Crisis in Italy: What We Know So Far, and Early Lessons. Planta Med..

[127] Donelli D., Antonelli M., Firenzuoli F. (2020). Considerations about Turmeric-Associated Hepatotoxicity Following a Series of Cases Occurred in Italy: Is Turmeric Really a New Hepatotoxic Substance?. Intern. Emerg. Med..

[128] Forsyth J.E., Nurunnahar S., Islam S.S., Baker M., Yeasmin D., Islam M.S., Rahman M., Fendorf S., Ardoin N.M., Winch P.J. (2019). Turmeric Means “Yellow” in Bengali: Lead Chromate Pigments Added to Turmeric Threaten Public Health across Bangladesh. Environ. Res..

[129] Forsyth J.E., Weaver K.L., Maher K., Islam M.S., Raqib R., Rahman M., Fendorf S., Luby S.P. (2019). Sources of Blood Lead Exposure in Rural Bangladesh. Environ. Sci. Technol..

[130] Chatzinasiou L., Booker A., MacLennan E., Mackonochie M., Heinrich M. (2019). Turmeric (*Curcuma longa* L.) Products: What Quality Differences Exist?. J. Herb. Med..

[131] Booker A., Frommenwiler D., Johnston D., Umealajekwu C., Reich E., Heinrich M. (2014). Chemical Variability along the Value Chains of Turmeric (*Curcuma longa*): A Comparison of Nuclear Magnetic Resonance Spectroscopy and High Performance Thin Layer Chromatography. J. Ethnopharmacol..

[132] Cao Y., Xu R.X., Liu Z. (2014). A High-Throughput Quantification Method of Curcuminoids and Curcumin Metabolites in Human Plasma via High-Performance Liquid Chromatography/Tandem Mass Spectrometry. J. Chromatogr. B Anal. Technol. Biomed. Life Sci..

[133] Song W., Qiao X., Liang W.F., Ji S., Yang L., Wang Y., Xu Y.W., Yang Y., Guo D.A., Ye M. (2015). Efficient Separation of Curcumin, Demethoxycurcumin, and Bisdemethoxycurcumin from Turmeric Using Supercritical Fluid Chromatography: From Analytical to Preparative Scale. J. Sep. Sci..

[134] Hong E., Lee S.Y., Jeong J.Y., Park J.M., Kim B.H., Kwon K., Chun H.S. (2017). Modern Analytical Methods for the Detection of Food Fraud and Adulteration by Food Category. J. Sci. Food Agric..

[135] Drivelos S.A., Georgiou C.A. (2012). Multi-Element and Multi-Isotope-Ratio Analysis to Determine the Geographical Origin of Foods in the European Union. TrAC Trends Anal. Chem..

[136] Friesen J.B., Liu Y., Chen S.N., McAlpine J.B., Pauli G.F. (2019). Selective Depletion and Enrichment of Constituents in “Curcumin” and Other *Curcuma longa* Preparations. J. Nat. Prod..

[137] Kim S.B., Bisson J., Friesen J.B., Bucchini L., Gafner S., Lankin D.C., Chen S.N., Pauli G.F., McAlpine J.B. (2021). The Untargeted Capability of NMR Helps Recognizing Nefarious Adulteration in Natural Products. J. Nat. Prod..

[138] Siudem P., Szeleszczuk Ł., Zielińska A., Paradowska K. (2023). ^13^C CPMAS NMR as an Alternative Method to Verify the Quality of Dietary Supplements Containing Curcumin. Molecules.

[139] Gafner S., Orhan N., Kahraman Ç., Blumenthal M. (2026). A Scoping Review of Turmeric Adulteration Based on Data from Six Continents. Pharm. Biol..

[140] You H., Gershon H., Goren F., Xue F., Kantowski T., Monheit L. (2022). Analytical Strategies to Determine the Labelling Accuracy and Economically-Motivated Adulteration of “Natural” Dietary Supplements in the Marketplace: Turmeric Case Study. Food Chem..

[141] Nounou M.I., Ko Y., Helal N.A., Boltz J.F. (2018). Adulteration and Counterfeiting of Online Nutraceutical Formulations in the United States: Time for Intervention?. J. Diet. Suppl..

[142] Mandal M., Jaiswal P., Mishra A. (2020). Role of curcumin and its nanoformulations in neurotherapeutics: A comprehensive review. J. Biochem. Mol. Toxicol..

[143] Askarizadeh A., Barreto G.E., Henney N.C., Majeed M., Sahebkar A. (2020). Neuroprotection by curcumin: A review on brain delivery strategies. Int. J. Pharm..

[144] Anand P., Kunnumakkara A.B., Newman R.A., Aggarwal B.B. (2007). Bioavailability of curcumin: Problems and promises. Mol. Pharm..

[145] Pan Y., Ju R., Cao X., Pei H., Zheng T., Wang W. (2020). Optimization extraction and purification of biological activity curcumin from *Curcuma longa* L by high-performance counter-current chromatography. J. Sep. Sci..

[146] Veldman B.A., Wijn A.M., Knoers N., Praamstra P., Horstink M.W. (1998). Genetic and environmental risk factors in Parkinson’s disease. Clin. Neurol. Neurosurg..

